# Community ecology and functional potential of bacteria, archaea, eukarya and viruses in Guerrero Negro microbial mat

**DOI:** 10.1038/s41598-024-52626-y

**Published:** 2024-01-31

**Authors:** P. Maza-Márquez, M. D. Lee, B. M. Bebout

**Affiliations:** 1 Exobiology Branch, NASA Ames Research Center, Moffett Field, CA USA; 2 University of Granada, Granada, Spain; 3 Blue Marble Space Institute of Science, Seattle, WA USA

**Keywords:** Biodiversity, Metagenomics, Microbiome, Evolutionary genetics, Ecological networks

## Abstract

In this study, the microbial ecology, potential environmental adaptive mechanisms, and the potential evolutionary interlinking of genes between bacterial, archaeal and viral lineages in Guerrero Negro (GN) microbial mat were investigated using metagenomic sequencing across a vertical transect at millimeter scale. The community composition based on unique genes comprised bacteria (98.01%), archaea (1.81%), eukarya (0.07%) and viruses (0.11%). A gene-focused analysis of bacteria archaea, eukarya and viruses showed a vertical partition of the community. The greatest coverages of genes of bacteria and eukarya were detected in first layers, while the highest coverages of genes of archaea and viruses were found in deeper layers. Many genes potentially related to adaptation to the local environment were detected, such as UV radiation, multidrug resistance, oxidative stress, heavy metals, salinity and desiccation. Those genes were found in bacterial, archaeal and viral lineages with 6477, 44, and 1 genes, respectively. The evolutionary histories of those genes were studied using phylogenetic analysis, showing an interlinking between domains in GN mat.

## Introduction

Microbial mats are one of the most ancient ecosystems known, having persisted through around 85% of the Earth’s history^1^ and played a key role in the evolution of Earth’s atmosphere^2,3^. Today’s mats are modern analogues of these first ecosystems on the Earth. As microbial mats were likely the locations at which oxygen was first produced in an otherwise anoxic world, they may offer an ecological model to understand both the evolution of biochemical cycles and microbial adaptation to drastic environmental changes, including the advent of an oxygenated atmosphere. Microbes found in microbial mats have been shown to exhibit a number of adaptive responses to extreme environmental conditions. In one of the few metagenomics studies of microbial mats, genes involved in adaptive responses and resilience against high-UV irradiation, elevated salinity conditions, oxidative stress and heavy metal resistance were described from microbial mats from Shark Bay^4^– one of the most extensive marine microbial mat systems in the world.

Guerrero Negro microbial mat is one of the best studied microbial mat ecosystems, and the use of molecular analysis based on sequencing of small-subunit rRNA genes and 454 sequencing have revealed the high complexity of this stratified ecosystem^5,6^. Mats located in salterns managed for the production of salt are permanently submerged by a hypersaline water column which serves to protect the vertical structure of the mats—relative to mats found in intertidal environments subjected to more profound environmental disturbance^7^. Earlier work detected a vertical stratification of oxygen and sulfide as well as a surprising occurrence of sulfate reduction in the oxic zone of the mat^8^. A recent study revealed a vertical patterns of nitrogen cycling genes with respect to vertical variations in oxygen concentration^9^. The vertical organization of other functions in these communities has been less well studied. GN microbial mats are characterized by extreme vertical chemical gradients at micrometer to millimeter spatial scales, and these vertical gradients have been shown to correspond to changes in microbial community composition^6^. Bacteria and archaea have been well documented in GN, as well as fungi in a recent study (within the eukarya domain)^10^. However, the composition and variability of viruses and their functions at fine scale resolutions in these communities is less known. Through metagenomics, the current study provides a taxonomic description of the vertical taxonomic organization as well as a functional organization delineated between bacteria, archaea, eukarya and viruses in a GN microbial mat—revealing new insights into the ecology of these communities*.* The goals of the present study were to characterize the community structure and their functional potential in this microbial mat through: (I) analyzing the functional annotations and taxonomic classification of assembled genes spanning bacterial, archaea, eukarya and viruses; (II) examining potential genetic mechanisms of adaptation in microbial mat; and (III) exploring the interlinkages between genes present in bacteria, archaea, eukarya, and viruses through gene-level phylogenetic analysis.

## Material and methods

### Microbial mat sampling

The sampled microbial mats are located in salterns managed by the world’s largest salt-producing company (Exportadora de Sal SA ESSA), situated on the Pacific Ocean side of the Baja peninsula. Exportadora de Sal has minimum human activity and consists of a series of 13 concentration areas with an approximate extension of 28,184 hectares. The concentration areas were constructed approximately 2 m above sea level in the low lands adjacent to the ponds “Ojo de Liebre” and “Guerrero Negro” (Fig. S1). The microbial mats were collected in June 2019 from hypersaline ponds concentration area 4 (Fig. S1), as previously described^10^. At the time of mat collection, salinity of brine was 125 ppt, temperature 24.4 C, pH 8.3, ammonium concentration 0.12 μM, dissolved oxygen 7 mg/L, and nitrate concentration was below the limit of detection (< 0.5 μM). Samples from the microbial mats were collected with a stainless-steel corer of 1 cm diameter as previously described^10^. Three replicate cores were placed into sterile centrifuge tubes (Falcon^®^, Corning, Corning, NY, USA), capped, and immediately frozen in liquid nitrogen. To get the vertical layers at one-millimeter intervals of the first four layers (0–1, 1–2, 2–3 and 3–4 mm from the top of the mat), the mat was sectioned using sterile scalpels. Three replicates were pooled for metagenomic analysis, resulting in a single pooled metagenome for each depth for library preparation and sequencing.

### DNA extraction

Total DNA extraction (from approximately 0.20 g per sample) was performed from each microbial mat layer, using a DNeasy Power Biofilm Kit, (Qiagen, Venlo, The Netherlands), according to manufacturer’s instructions. A nanophotometer (Implen GmbH, München, Germany) was used for checking the quality (A260/A280) and quantity (A260) of extracted genomic DNA. Library preparation and metagenomic sequencing were performed at Molecular Research (MR DNA, Texas, USA, http://www.mrdna.org/contact.html). Libraries were prepared using the Nextera DNA Flex library kit (Illumina) following the manufacturer’s instructions, and sequencing was performed on the NovaSeq 6000 platform (2 × 150 nucleotides).

### Metagenomic data processing

Metagenomics processing with annotated code is documented at our open-Science Foundation site, https://osf.io/9kwn3/wiki^11^. Conda (2020; www.anaconda.com (accessed on 10 March 2021)) was utilized for program installation and environment management. Read quality was scanned with FastQC v0.11.9^12^ and reads trimmed/filtered with trimmomatic v0.39^13^. A co-assembly of all 4 depths was performed with SPAdes v3.14.0^14^, the assembly was filtered and summarized with bit v1.8.16^15^ (see Table S1 for assembly summary statistics), and each individual samples’ reads were mapped to the filtered co-assembly with bowtie2 v2.3.5.1^16^ and sorted and indexed with samtools v1.9^17^. Metagenomic sequence data from the 4 depths are available through NCBI’s Sequence Read Archive at BioProject PRJNA688760. NCBI accession numbers: SRX9761389, SRX9761388, SRX9761387 and SRX9761386.

#### Taxonomic classification and functional annotation

Co-assembly and read-mapping files were integrated into anvi’o v6.2^18^ for annotation with the KEGG database^19^ and parsing and extraction of the gene-level coverage and detection data (with “detection” being the proportion of a gene that recruited any reads to it). Gene-level taxonomic classification was performed with CAT v5.1.2^20^ against the NCBI nr database. Normalization and analyses were performed with R v3.6.3 (Core Team 2017) in Rstudio v1.1.456 (www.rstudio.com). To mitigate non-specific read-recruitment, gene-level coverage information was filtered based on detection (proportion of a gene that recruited any reads to it), such that those with a detection less than 50% had their coverage set to 0. This had a net effect of removing less than 3% of the pre-filtered total coverage.

Information on KEGG’s Metabolism pathway was accessed with KEGGREST v1.26.0, defining the KEGG Orthology (KO) terms. The gene table coverages were normalized across the 4 samples by dividing each value by its sample’s total coverage and multiplying by 1 million to generate values of Coverage per Million (herein referred to as CPM). Entrez Direct v13.9, www.ncbi.nlm.nih.gov/books/NBK179288/) was utilized to search and retrieve reference sequences from NCBI. Heatmap and cluster analyses based on Bray Curtis similarities were calculated for all genes with a mean coverage of >= 9 in when summed across the 4 samples.

#### Tree construction

For making phylogenetic trees, sequences were aligned with Muscle v3.8.1551^21^, and then trimmed using trimal v1.4.1. Trees were generated using the open-source software IQ-TREE v2.2.0^22^. We inferred the maximum-likelihood tree with auto-model selection via the built-in ModelFinder (option `-m MFP`) using 1000 bootstraps^23^. The trees were edited through the Interactive Tree of Life web-interface^24^. Accurately rooting is essential for the correct interpretation of the genetic changes between sequences since the root gives the directionality of evolution within the tree^25^. However, due to the span of diversity we were considering (across all domains), and to have a consistent method of rooting across all the generated gene trees, we utilized mid-point rooting^26^. All trees in Newick format are archived with 10.6084/m9.figshare.25018007 and available here: https://doi.org/10.6084/m9.figshare.25018007.

#### Viral analysis

For viral specific identification, contigs from the initial co-assembly were screened for viral sequences using VIRSorter2 v3, then checkV was passed for quality control of the VirSorter2 results, following previous protocols^27^. Viral read of each sample were mapped using bowtie2 v2.3.5.1^16^ and sorted and indexed with samtools v1.9^17^. Anvi’o v6.2^18^ was used to annotate viral gene and calculate coverage profiles. For virus gene level taxonomic classification, two databases were used: RefSeq84^28^ and IMGVR^29^. To avoid false positive viral gene identification, the genes were filtered based on two criteria: genes taxonomic classified in the RefSeq84 and/or IMGVR databases; and functional viral protein annotated in KEGG database.

## Results

### A gene-focused view of bacteria, archaea, eukarya, and viruses.

As described in Methods, metagenomes from all 4 depths were co-assembled together, genes were identified, and normalized coverage values were attained by recruiting the individual sample reads to the assembled contigs. Mean coverage values for genes were extracted, and for exploratory purposes these mean coverage values for each sample were normalized to be out of 1 million (coverage per million (CPM)). Genes identified were taxonomically classified and functionally annotated, and here we break down those results. We also include information on read-based classification, as a means to potentially identify if any large biases might have been introduced through the assembly-to-gene-calling process, but the results did not largely vary in any cases.

#### A gene-focused view of Bacteria

**Figure 1:**
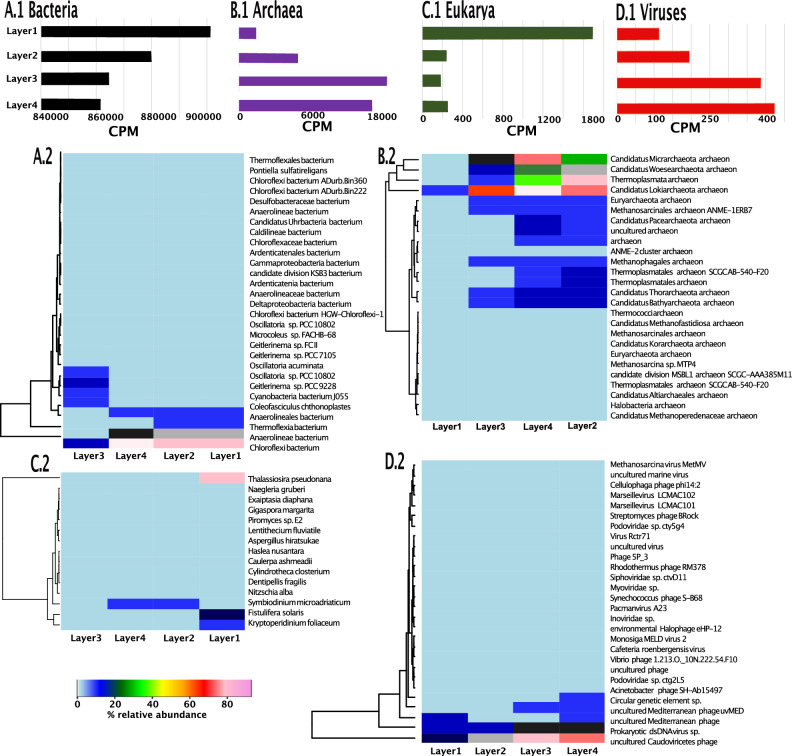
Summaries of normalized gene-level coverages broken down by domain; note the x-axes vary between A1-D1. (**A1**) Bar plots of the genes identified in bacteria across the uppers 4 layers examined [Layer 1 (0-1 mm from surface), Layer 2(1–2 mm from surface), Layer3 (2–3 mm from surface), and Layer 4 (3-4 mm from surface)]. (**A2**) Heatmap showing the read-based taxonomic classification of bacteria at species level with coverages >= 9 across the uppers 4 layers examined. (**B1**) Bar plots of the CPM of the 14,184 genes identified in archaea across the uppers 4 layers examined [Layer 1 (0–1 mm from surface), Layer 2(1–2 mm from surface), Layer 3 (2–3 mm from surface), and Layer 4 (3–4 mm from surface)]. (**B2**) Heatmap showing the read-based taxonomic classification of archaea at species level across the uppers 4 layers examined. (**C1**) Bar plots of the CPM of the 547 genes identified in eucaryote across the uppers 4 layers examined [Layer 1 (0–1 mm from surface), Layer 2(1–2 mm from surface), Layer 3 (2–3 mm from surface), and Layer 4 (3–4 mm from surface)]. (**C2**) Heatmap showing the read-based taxonomic classification of eucaryotes at species level across the uppers 4 layers examined. (**D1**) Bar plots of the CPM of the 394 genes identified in viruses across the uppers 4 layers examined [Layer 1 (0–1 mm from surface), Layer 2(1–2 mm from surface), Layer 3 (2–3 mm from surface), and Layer 4 (3–4 mm from surface)]. (**D2**) Heatmap showing the read-based taxonomic classification of viruses at species level across the uppers 4 layers examined.

Taxonomically classified within the bacterial domain, 764,307 unique genes were assembled and identified, with 461,332, 679,559, 656,264 and 677,165 of them having a coverage greater than 0 in Layer 1, Layer 2, Layer 3, and Layer 4, respectively (Tables S2 and S3; Fig. 1A1). However, the total normalized coverages of the bacterial genes decreased with depth (Fig. 1A1). Layer 1 contained the greatest coverage of bacterial genes, with 901,325.3 CPM, while Layers 3 and Layer 4 contained the lowest, 864,618.79 and 861,536.72 CPM, respectively. Heatmap and cluster analyses based on Bray Curtis resemblance of the genes are shown in Fig. S2A1. The global similarity was > 40%, with Layer 1 being the least similar and clustering out separately; the similarity between Layer 2, Layer 3, and Layer 4 was > 80%. SIMPROF analysis based on Bray Curtis resemblance at 5% of significance level detected a significant difference between Layers 3 and Layer 4 (*p* = 0.001) and Layer 3-Layer 4, and Layer 2 (*p* = 0.001), and between Layer 3-Layer 4- Layer 2 and Layer 1 (*p* = 0.001) 32.8% of bacterial genes were successfully functionally annotated with KO terms, comprising 357,984 ± 13,375 CPM across the 4 layers (mean ± 1SD; Table S2). According to KEGG’s groupings, genes with >= 9 average CPM across the 4 layers were ascribed to 42 metabolic pathways, with the most abundant being related to genetic information processing, signaling and cellular processes, carbohydrate metabolism, and energy metabolism (Table S2).

Gene-level taxonomic classification within the Bacteria detected 44 phyla, 106 families, and 430 species (Fig. 1A2, S3, Table S3). The dominant phyla were Cyanobacteria, followed by Chloroflexi, Proteobacteria, Firmicutes and Bacteroidetes. Cyanobacteria were dominant in Layer 1, while Chloroflexi was dominant in Layer 4. At family level, *Microcoleaceae* was the most prevalent family, followed by *Coleofasciculaceae* and *Oscillatoriaceae*. At species level, the dominant species were *Chloroflexi* bacterium, *Anaerolineae* bacterium, *Anaerolineales* bacterium, *Geitlerinema* sp. PCC 9228 and *Cyanobacteria* bacterium J055. *Chloroflexi* bacterium, *Geitlerinema* sp. PCC 9228, *Cyanobacteria* bacterium J055 and *Coleofasciculus chthonoplastes* were the predominant species in Layer 1, while *Chloroflexi* bacterium, *Anaerolineae* bacterium, *Anaerolineales* bacterium and *Thermoflexia* bacterium were dominant species in Layer 4.

#### A gene-focused view of Archaea

For the archaeal domain, 14,148 unique genes were identified with 2,762, 8,338, 13,250, 13,793 having coverages > 0 for Layer 1, Layer 2, Layer 3, and Layer 4, respectively. The highest coverages of genes were detected in Layer 3 and 4 (Fig. 1B1), which were > 7 times more abundant than in Layer 1. The dendrogram in Fig. S2A2 shows the heatmap and cluster analyses of samples based on Bray Curtis similarities of the archaeal composition of the community. Layers 3 and 4 were more similar overall, at > 70% similarity, while Layer 1 was the least similar (Fig. S2A2). According to SIMPROF analysis, there was a significant difference in the archaeal gene coverages (based on an alpha value of 0.05) between Layer 3 and Layer 4 (*p* = 0.022), Layers 3- Layer 4 and Layer 2 (*p* = 0.001), and Layers 3- Layer 4- Layer 2 and Layer 1 (*p* = 0.001). 25.7% of archaeal genes were successfully functionally annotated with KO terms, with those unannotated accounting for 7,792.4 ± 5,215.8 CPM across the depths (Table S4). The 14,148 genes were classified into 26 KEGG metabolic pathways (Table S4). Genetic information processing was the most abundant pathway, while the lowest abundant pathways were related to amino acids, cellular processes, cell motility, and sulfur metabolisms (Table S4).

Gene-level taxonomic classification of archaea is summarized in Figs. 1B2, S4 and Table S5. A total of 20 phyla, 36 families and 496 species were identified. The dominant phyla were Euryarchaeota, followed by Candidatus Lokiarchaeota, Candidatus Micrarchaeota, Candidatus Woesearchaeota, Candidatus Thorarchaeota. At family level, *Methanosarcinaceae* were the dominant family, followed by *Methanotrichaceae, Methanobacteriaceae* and Candidatus *Methanoperedenaceae*. At species level, Candidatus *Lokiarchaeota* archaeon were the dominant species, followed by *Thermoplasmata* archaeon, Candidatus *Micrarchaeota* archaeon and Candidatus *Woesearchaeota* archaeon.

#### A gene-focused view of Eukarya

For the eukaryal domain, 547 genes were detected, with 403, 396, 298, 318 of them having coverages > 0 for Layer 1, Layer 2, Layer 3 and Layer 4, respectively. The greatest coverages were identified in Layer 1, with 1,696.08 CPM (Fig. 1C1). The coverages in Layers 2–4 were > 6 times lower than in Layer1, with 241.6, 188.6, 257.7 CPM for Layer 2, 3 and 4, respectively. The heatmap and cluster analysis representing the similarity of the 547 genes with depth is show in Fig. S2A3. The global similarity of the eukaryal community was low, < 5%. Layer 1 represented the least similar one, while the similarity between Layers 3 and 4 was > 80%. According to SIMPROF analysis, there was a significant difference in the coverage (based on an alpha value of 0.05) between Layer 1 and Layer 2-Layer 3-Layer 4 (*p* = 0.001), and between Layer 2-Layer 3 and Layer 4 (*p* = 0.001), but not between Layer 3 and Layer 4 (*p* = 1), where the similarity was > 80%. 26.7% of eukaryal genes were successfully functionally annotated with KO terms, with those unannotated accounting for 377.6 ± 352.6 CPM across the depths (Table S6). According to KEGG orthology classification, the 547 genes were grouped in 11 metabolic pathways (Table S6). The most abundant pathways were genetic information processing and photosynthesis. Overwise, the minor coverages of genes were related to the pathways sulfur relay system and quorum sensing (Table S6).

Gene-level taxonomic classification of eukarya identified 19 phyla, 119 families and 15 species (Fig. 1C2, S5, Table S7). Bacillariophyta were the dominant phyla, followed by Streptophyta and Ascomycota. At family level, *Thalassiosiraceae* were the most predominant family, followed by *Symbiodiniaceae* and *Bacillariaceae.* At species level, *Thalassiosira pseudonana* was the predominant species, followed by *Symbiodinium microadriaticum* and* Fistulifera solaris.*

#### A gene-focused view of Viruses

835 genes were assembled and taxonomically classified as viral, with 451, 474, 791 and 759 of them having a coverage greater than 0 in Layers 1, 2, 3, and 4, respectively. The coverages of viral genes increased with depth, with Layers 3 and 4 having coverage values > 3 times higher than in Layer 1 (Fig. 1D1). Figure S2A4 shows the heatmap and cluster analysis based on the coverages of the 835 genes identified. The global similarity between the layers was < 20%, with Layer 1 again being the least similar. A SIMPROF test at 5% of significance level revealed no significance difference in the coverage of the genes between Layer 3 and Layer 4 (*p* = 0.35), but detected significant differences between Layers 3-Layer 4 and Layer 2 (*p* = 0.001), and between Layer 2-Layer 3- Layer 4 and Layer 1 (*p* = 0.001). 17.9% of identified viral genes were successfully functionally annotated with KO terms, with those unannotated accounting for 1531.0 ± 1095.9 CPM across the depths (Table S8). KEGG grouped these genes into 10 pathways (Table S8), the highest coverages were related to genetic information processing and phage terminase large subunit/ phage replication initiation protein.

Figure S6 shows the read-based classification of the identified virome at phyla and family levels: 3 phyla, 16 families and 149 species were identified (Table S9). The dominant phyla were Uroviricota, followed by Nucleocytoviricota and Hofneiviricota. At species level, the most abundant species was related to uncultured *Caudoviricetes* phage, following by Prokaryotic dsDNA virus sp., uncultured *Mediterranean* phage uvMED, uncultured *Mediterranean* phage, *Streptomyces* phage Brock, *Cellulophage* phage phi17:1, *Podoviridae* sp. cty5g4, *Marseillevirus* LCMAC101 (Fig. 1D2).

### Living in Guerrero Negro microbial mat: bacteria, archaea, and virus genes related to potential adaptation mechanisms

**Figure 2:**
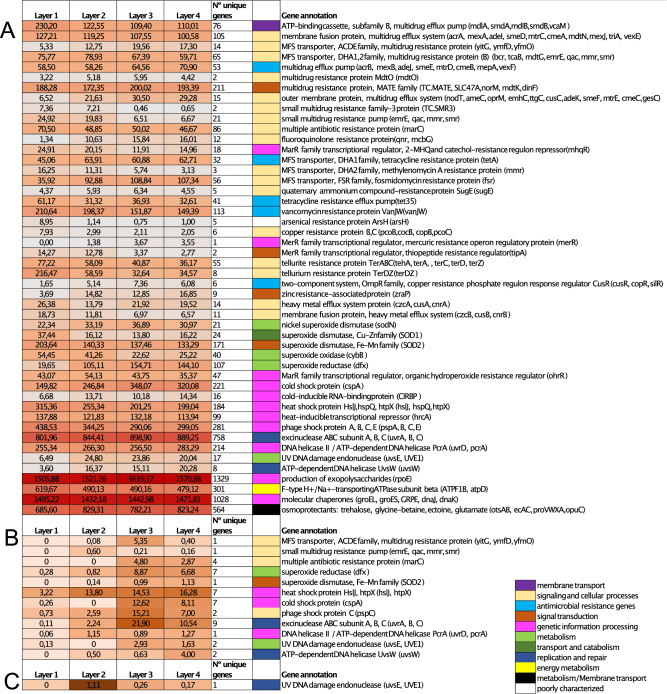
(**A**) Heatmap showing the coverages of potentially adaptation-relevant genes present in bacteria across the uppers 4 layers examined. (**B**) Heatmap showing the coverages of potentially adaptation-relevant genes present in archaea across the uppers 4 layers examined. (**C**) Heatmap showing the UV gene present in virus across the uppers 4 layers examined.

Here we focus on recovered genes related to antibiotic and multidrug resistance, heavy metal toxicity, oxidative damage genes, cold, heat and phage shock proteins, UV-radiation stress genes, salinity and desiccation stress conditions. We recovered 6477 unique genes annotated with those functions that were classified within the bacterial domain and 44 within the archaeal domain (Fig. 2, Tables S10, S11, S12, S13, S14, S15, S16 and S17). Moreover, one gene (related to UV-DNA damage endonuclease Table S18) was recovered that was classified as originating from a virus. According to KEGG, those genes were classified within membrane transport (76), signaling and cellular processes (488), antimicrobial resistance genes (245), signal transduction (394), genetic information processing (4220), metabolism (758), transport and catabolism (24), replication and repair (10) and energy metabolism (301) (Fig. 2). The presence of these genes may have implications for adaptability and resilience to stress conditions, as has been previously described in another microbial mat^4^.

#### Genes related to potential adaptation mechanism in Bacteria

For Bacteria, 6477 genes related to potential environmental adaptation were detected (Fig. 2A and supplementary Tables S10–S16). According to KEGG orthology classification, the 6477 genes were classified within membrane transport (76), signaling and cellular processes (480), antimicrobial resistance genes (245), signal transduction (393), genetic information processing (3834), metabolism (749), transport and catabolism (24), replication and repair (370) and energy metabolism (301): 564 unique genes were related to multidrug resistance protein/multidrug efflux pump (Table S10); 86 unique genes were detected for multiple antibiotic resistance protein; 280 genes were detected for fluoroquinolone, catechol, tetracycline, fosmidomycin, quaternary compound, tetracycline and vancomycin resistance protein; 92 unique genes were identified to resistance to arsenical, copper, mercuric, tellurite and zinc (Table S11); 25 genes were related to heavy metal (Table S12); 410 involved in dealing with oxidative stress (Table S13); 237 genes were related to cold shock proteins (Table S14); 283 genes were identified as heat shock proteins (Table S14); 281 genes for phage shock protein (Table S14); 997 genes were related to UV damage: excinuclease ABC subunit ABC, DNA helicase II /ATP-dependent DNA helicase PcrA, UV DNA damage endonuclease, and ATP-dependent DNA helicase UvsW (Table S15).; and 3222 genes were associated with desiccation and salinity conditions (Table S16): 1329 genes were annotated as RNA polymerase sigma-70 factor, ECF subfamily (*rpoE*), 301genes for F-type H + /Na + -transporting ATPase subunit beta (*ATPF1B, atpD*), 281 genes for chaperonin GroEL (*groEL, HSPD1*), 251 genes for chaperonin GroES (*groES, HSPE1*), 259 genes for molecular chaperone GrpE (*GRPE*), 121 genes for molecular chaperone DnaJ (*dnaJ*), 116 genes for molecular chaperone DnaK (*dnaK, HSPA9*), 15 genes for trehalose 6-phosphate synthase (*otsA*), 35 genes for trehalose 6-phosphate phosphatase (*otsB*), 81 genes for osmoprotectant transport system substrate-binding protein (*opuC*), 113 genes for glycine betaine/proline transport system ATP-binding protein (*proV*), 106 genes for glycine betaine/proline transport system permease protein (*proW*), 206 genes for glycine betaine/proline transport system substrate-binding protein (*proX*) and 8 genes for L-ectoine synthase (*ectC*). Summary of these genes and their normalized coverages are showed in Fig. 2A.

Overall, layer 1 contained the highest coverages of genes involved in antibiotic and multidrug resistance (pumps ATP-binding cassette, subfamily B, multidrug efflux pump and membrane fusion protein, multidrug efflux system), resistance to metals (arsenic, copper resistance protein, tellurite, tellurium and tetracycline genes), heavy metal, oxidative stress genes (superoxide dismutase, Cu–Zn family, superoxide dismutase Fe, Mn family and superoxide oxidase) and phage and heat shock proteins. On the other hand, deeper layers contained the greatest coverages of genes related to multidrug resistance genes (MFS transporter, ACDE family, multidrug resistance protein, multidrug resistance protein, MATE family and outer membrane protein, multidrug efflux system), zinc resistance gene, superoxide reductase gene, cold shock protein and genes associated with UV-resistance/repair (*uvrA, uvrB, uvrC* and *uvrD* genes). Genes involved with desiccation and salinity stress conditions had similar coverages across layers, detecting the greatest coverages for genes to encoded the production of exopolysaccharides (*rpoE*) and molecular chaperones (*GroES, GroEL, DnaJ* and *DnaK*). Deeper discussion to explain their relevance in the context of potential ecological adaptations of microbial mats is detailed in Section “Discussion”.

#### Genes related to potential adaptation mechanism in Archaea

In the archaeal domain, 44 genes identified in the metagenome data were related to stress conditions: 2 for multidrug resistance, 4 for multiple antibiotic resistance, 8 for interactions with oxidative genes, 16 shock proteins and 14 for UV genes (Fig. 2B, Table S17). Layer 3 and 4 contained the greatest coverages of multidrug resistance genes, antibiotic, oxidative genes (superoxide reductase and superoxide dismutase), cold and phage shock proteins and UV genes. On the other hand, antibiotic resistance genes were not recovered in Layer 1 or 2; while heat shock protein *hsIJ* was higher than *htpX* and the greatest coverages were found in Layer 2. According to KEGG orthology classification, the 44 genes were classified within signaling and cellular processes (8), signal transduction (1), genetic information processing (18), metabolism (9), replication and repair (8). Deeper discussion to explain their relevance in the context of potential ecological adaptations of microbial mats is detailed in Section “Discussion”.

### Interlinking between the genes present in bacteria, archaea and viruses using phylogenetic analysis

**Figure 3:**
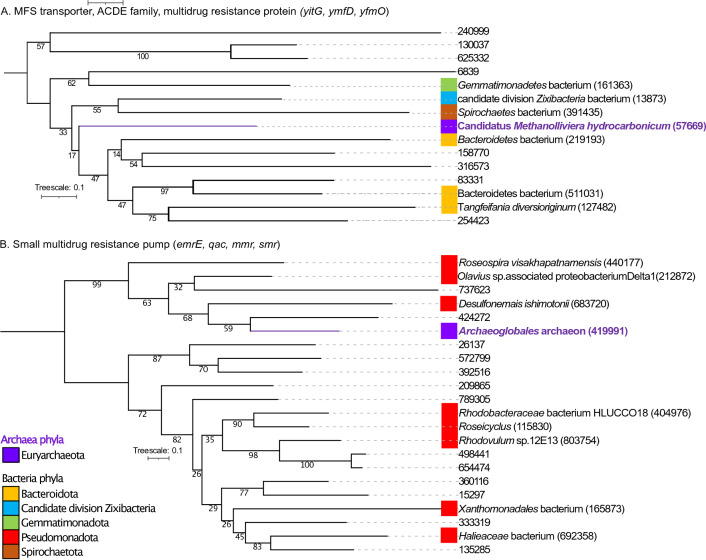
(**A**) Mid-point rooted phylogenetic tree based on amino-acid sequences detected in our study from MFS transporter, ACDE family, multidrug resistance protein (*yitG*, *ymfD*, *yfmO*). (**B**) Phylogenetic tree based on amino-acid sequences from small multidrug resistance pump (*emrE, qac, mmr, smr*). Amino-acid sequences from bacteria (black color), amino acid sequences from archaea (purple color). For built the tree, 37 sequences were included in the phylogenetic analysis (15 sequences for *yitG, ymfD, yfmO* genes and 22 sequences for *emrE, qac, mmr, smr* genes). The sequences were aligned with Muscle and the tree was generated with IQTREE2 with 1000 bootstraps, with auto-model selection via the built-in ModelFinder.

**Figure 4:**
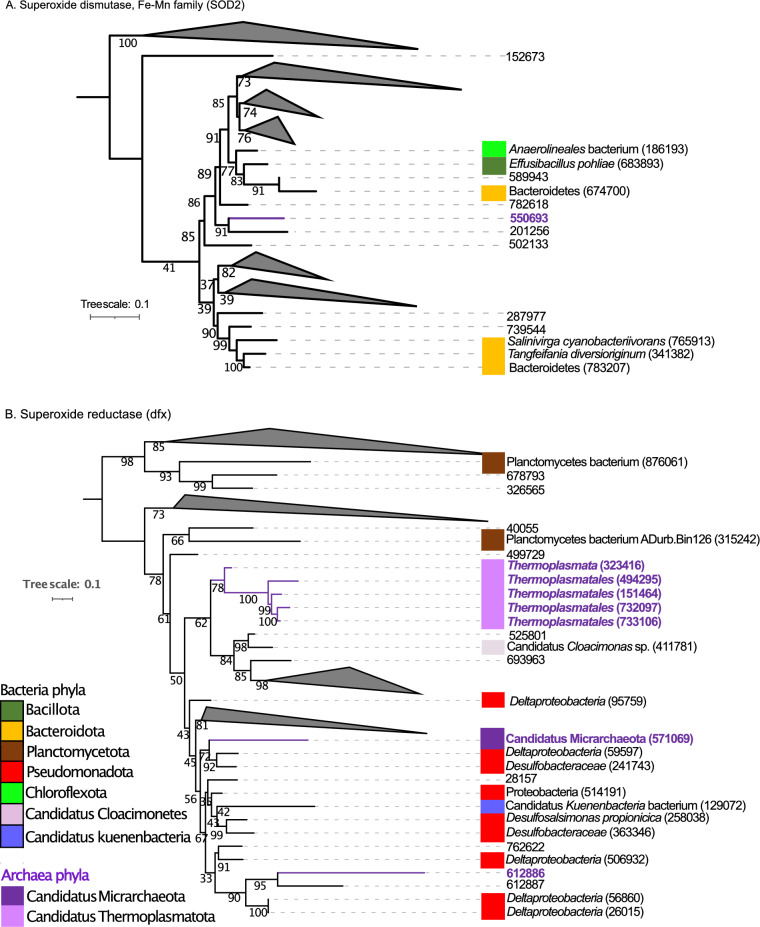
(**A**) Mid-point rooted phylogenetic tree based on amino-acid sequences detected in our study from superoxide dismutase, Fe–Mn family (*SOD2*). (**B**) Phylogenetic tree based on amino-acid sequences from superoxide reductase (*dfx*). Amino-acid sequences from bacteria (black color), amino acid sequences from archaea (purple color). For built the tree, 286 sequences were included in the phylogenetic analysis (172 sequences for *SOD2* gene and 114 sequences for *dfx* gene). The sequences were aligned with Muscle and the tree was generated with IQTREE2 with 1000 bootstraps, with auto-model selection via the built-in ModelFinder.

**Figure 5:**
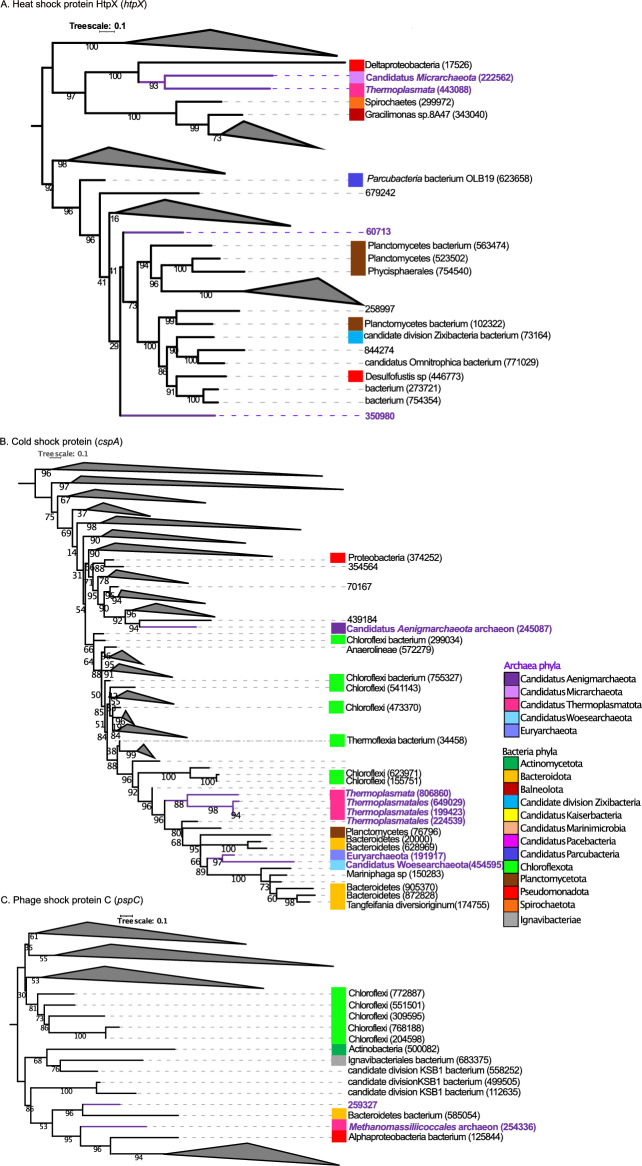
(**A**) Mid-point rooted phylogenetic tree based on amino-acid sequences detected in our study from heat shock protein HtpX (*htpX*). (**B**) Phylogenetic tree based on amino-acid sequences from cold shock protein (*cspA*). (**C**) Phylogenetic tree based on amino-acid sequences from phage shock protein C (*pspC*). Amino-acid sequences from bacteria (black color), amino acid sequences from archaea (purple color). For built the tree, 454 sequences were included in the phylogenetic analysis (110 sequences for *htpX* gene, 228 sequences for *cspA* gene and 116 sequences for *pspC* gene). The sequences were aligned with Muscle and the tree was generated with IQTREE2 with 1000 bootstraps, with auto-model selection via the built-in ModelFinder.

**Figure 6:**
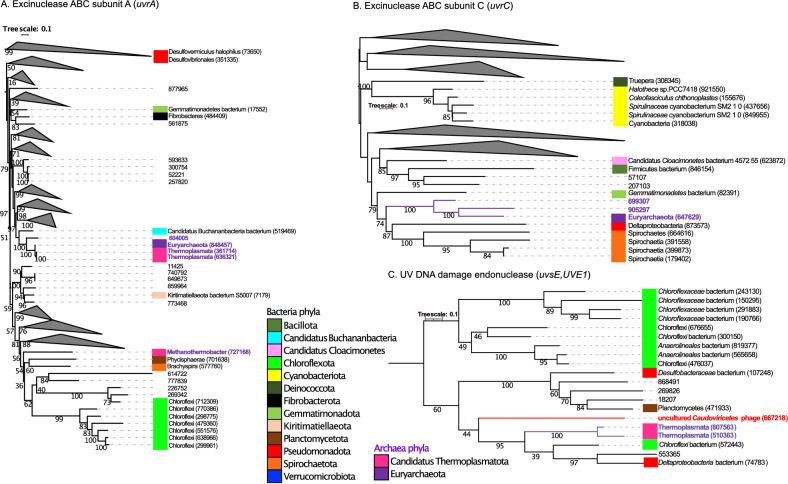
(**A**) Mid-point rooted phylogenetic tree based on amino-acid sequences detected in our study from excinuclease ABC subunit A (*uvrA*). (**B**) Phylogenetic tree based on amino-acid sequences from excinuclease ABC subunit C (*uvrC*). (**C**) Phylogenetic tree based on amino-acid sequences from UV DNA damage endonuclease (*uvsE, UVE1*). Amino-acid sequences from bacteria (black color), amino acid sequences from archaea (purple color), amino-acid sequences from viruses (red color). For built the tree, 593 sequences were included in the phylogenetic analysis (367 sequences for *uvrA* gene, 206 sequences for *uvrC* gene and 20 sequences for *uvsE, UVE1* gene). The sequences were aligned with Muscle and the tree was generated with IQTREE2 with 1000 bootstraps, with auto-model selection via the built-in ModelFinder.

To further examine the distribution of genes related to potential adaptative mechanisms in terms of their evolutionary relatedness, we built phylogenetic trees. A total of 12 trees were built for the genes: MFS transporter, ACDE family, multidrug resistance protein (KO ID K08221), small multidrug resistance pump (KO ID K03297), superoxide dismutase, Fe–Mn family (KO ID K04564), superoxide reductase (KO ID K05919), heat shock protein (KO ID K03799), cold shock protein (KO ID K03704), phage shock protein (KO ID K03973), excinuclease ABC subunit A (KO ID K03701), excinuclease ABC subunit B (KO ID K03702), excinuclease ABC subunit C (KO ID K03703), DNA helicase II ATP dependent DNA helicase (KO ID K03657) and UV DNA damage endonuclease (KO ID K13281). These genes were present in bacteria, archaea and viruses domains as follow: for KO ID K08221: 14 sequences were present in bacteria and 1 in archaea; for KO ID K03297: 21 sequences were detected in bacteria and 1 in archaea; for KO ID K04564: 171 sequences were present in bacteria and 1 in archaea; for KO ID K05919: 107 sequences were found in bacteria and 7 in archaea; for KO ID K03799: 106 sequences were present in bacteria and 4 in archaea; for KO ID K03704: 221 sequences were found in bacteria and 7 in archaea; for KO ID K03973: 114 sequences were present in bacteria and 2 in archaea; for KO ID K03701: 362 sequences were present in bacteria and 5 in archaea; for KO ID K03702: 193 sequences were present in bacteria and 1 in archaea; for KO ID K03703: 203 sequences were found in bacteria and 3 in archaea; for KO ID K03657: 214 were identified in bacteria and 1 in archaea; for KO ID K13281: 17 sequences were detected in bacteria, 2 in archaea and 1 in viruses. Figures 3, 4, 5 and 6 show the phylogenetic relationships between domains; the complete trees are in the supplementary material Figs. S7–S15. And see Table 1 and the corresponding supplemental tables for gene IDs and full amino-acid sequences.

Figure 3 shows the phylogenetic tree of MFS transporter, ACDE family, multidrug resistance protein (*yitG, ymfD, yfmO*; 3A) and small multidrug resistance pump (*emrE, qac, mmr, smr*; 3B). For ACDE family, multidrug resistance protein (*yitG, ymfD, yfmO*) (Fig. 3A), the gene ID 57669 present in archaea belonging to Candidatus *Methanolliviera hydrocarbonicum* was between clades holding genes classified as sourced from the following bacterial lineages: *Spirochaetes* bacterium (gene ID 391435), Candidate division *Zixibacteria* bacterium (gene ID 13873), *Bacteroidetes* bacterium (genes IDs 219193, 511031), *Tangfeifania diversioriginum* (gene ID 127482) and the genes IDs 158770, 316573, 83331 and 254423. With respect small multidrug resistance pump (*emrE, qac, mmr, smr*) (Fig. 3B), the gene ID 419991 present in *Archaeoglobales* archaeon formed a clade with 4 genes present in bacteria: the genes IDs 424272, 737623, gene ID 683720 present in *Desulfonemais himotonii* and gene ID 212872 present in *Olavius* sp. associated proteobacterium Delta1.

Figure 4 and Fig. S7–S8 show the phylogenetic trees for superoxide dismutase, Fe–Mn family (*SOD2*) (Fig. 4A and S7) and superoxide reductase (*dfx*) (Fig. 4B and S8). For SOD2, the gene ID 550693 present in archaea formed a clade with a gene ID 201256 present in bacteria. For *dfx* gene, the gene ID 612886 present in archaea formed a clade with 3 genes present in bacteria lineages: two present in Deltaproteobacteria (genes IDs 56860, 26015) and one present in bacteria gene ID 612887. The gene ID 571069 present in Candidatus *Micrarchaeota* was closely related to genes present in Deltaproteobacteria (gene ID 59597) and *Desulfobacteraceae* (gene ID 241743). Finally, 5 genes present in archaea with genes IDs 323416, 494295, 151464, 732097, 733106 presents in *Thermoplasmatales* and *Thermoplasmata* formed a clade. This clade was closely related to a clade formed by the bacterial lineages Candidatus *Cloacimonas* sp. (411781), *Peptoclostridium litorale* (685247), Clostridia (847882), Planctomycetes bacterium (315242) and the gene IDs 40055, 525801, 693963, 411780, 6209.

Figure 5 and Figures S9–S11 show the phylogenetic trees corresponding to heat, cold and phage shock protein. For heat shock protein HtpX (*htpx*) (Fig. 5A and S9), the gene IDs 443088 and 222562 from the archaea lineages of *Thermoplasmata* and Candidatus *Micrarchaeota* formed a clade with a gene recovered from a *Deltaproteobacteria* (gene ID 17526). The genes IDs 350980 and 60713 present in archaea deeply branched in between clades holding bacterial genes from Planctomycetes (563474, 523502, 102322), *Phycisphaerales* (754540), candidate division Zixibacteria (73164) and Desulfofustis sp (446773). Regarding to cold shock protein (*cspA*) (Fig. 5B and S10), the genes ID 806860, 245087, 649029, 199423 and 224539 detected from the archaeal lineages of *Thermoplasmata*, Candidatus *Aenigmarchaeota* archaeon and *Thermoplasmatales* formed a clade with two genes present in *Chloroflexi* bacterium (genes IDs 641093, 589131, 623971, 155751). The genes ID 454595 and 191917 presents in Candidatus *Woesearchaeota* and *Euryarchaeota* were in a deeply branching clade with genes from Planctomycetota (gene ID 76796) and Bacteroidetes (genes IDs 20000, 628969). For phage shock protein C (*pcpc*) (Fig. 5C and S11), the gene ID 259327 present in archaea formed a clade with genes from Bacteroidetes bacterium (gene ID 585054). The gene ID 254336 present in *Methanomas siliicoccalesa* formed a clade with bacterial genes from Alphaproteobacteria bacterium (gene ID 125844) and Bacteroidetes bacterium (gene ID 585054).

The phylogenetic trees representing the UV genes (*uvrA*, *uvrB, uvrC, uvrD, pcrA, uvsE, UVE1*) are shown in Fig. 6 and in the supplementary Figs. S12–S15. For *uvrA* gene (Fig. 6A and S12), three genes classified as coming from the archaeal lineages of Euryarchaeota and *Thermoplasmata* (genes IDs 848457, 361714, 636321) formed a clade with a bacteria gene ID 519469 from Candidatus Buchananbacteria bacterium. One gene classified as coming from *Methanothermobacter* (gene ID 727168) was within a deep clade with bacterial genes from Phycisphaerae (gene ID 701638) and Brachyspira (gene ID 577760). For *uvrB* gene (Fig. S13), the gene ID 653100 taxonomically classified as *Thermoplasmata* branched near genes classified as Planctomycetota (genes IDs 728796, 54766). For *uvrC* gene (Fig. 6B and S14), the genes IDs 699307, 905297, 647629 corresponding to archaea formed a clade with 6 genes present in bacterial lineages: Pseudomonadota (gene ID 873573), *Spirochaetes* (gene ID 664616), *Spirochaetia* (gene ID 391558), *Spirochaetia* (gene ID 399873), *Spirochaetia* (gene ID 179402) and *Gemmatimonadetes* bacterium (gene ID 82391). For *uvrD, pcrA* gene (Fig. S15), the gene ID 181004 present in Candidatus *Bathyarchaeota* formed a clade with a gene ID 297131 present in *Bacteroidales* bacterium*.* For *uvsE, UVE1* gene (Fig. 6C), two genes present in *Thermoplasmata* (genes IDs 807563 and 510363) and one gene present in uncultured *Caudoviricetes* phage (gene ID 667218) formed a clade with 3 genes present in bacteria (genes IDs 572443, 553365 and 74783, representing a *Chloroflexi* bacterium, an unclassified bacterium, and a *Deltaproteobacteria* bacterium, respectively).

## Discussion

Guerrero Negro microbial mat is one of the best studied microbial mat ecosystems; however, the vertical functional organization has been less well studied. In this study, 922,765 unique gene-copies were recovered (meaning assembled and predicted), with 84.51% of those being classified to at least the domain level, leaving 15.49% unclassified (Table S19). The greatest coverages of bacteria and eukarya genes were detected in Layer 1, while the highest coverages of viruses and archaea genes were found in Layers 3 and 4 (Fig. 1). The upper one-millimeters (Layer 1) was dominated by prokaryote Cyanobacteria (including the families of *Microcoleacea* and *Oscillatoriaceae*) and eukarya Bacillariophyta (Fig. 1, S3, S5). Layer 1 is mostly oxygenic and generally dominated by oxygenic phototrophs. Members of Bacillariophyta have been previously identified in phototrophic microbial mat in Iceland using 18S rDNA^30^. In this layer, the genes with highest normalized coverages were annotated as photosystem, for Cyanobacteria and Bacillariophyta, respectively. For Cyanobacteria, the highest coverages of genes were related to photosystem II P680 reaction center D2 protein (*psbD*) and photosystem II oxygen-evolving enhancer protein 3 (*psbQ*). Similarly, oxygenic photosynthesis genes have been identified from Cyanobacteria in a Shark Bay microbial mat in Australia^4^. Layer 1 also contained the greatest coverages of genes and the dominant phylum was Bacillariophyta (within Eukarya domain). The most abundant pathways for genes classified as eukaryal were related to genetic information processing and photosynthesis, with the greatest coverages coming from genes related to photosystem (psbX, psaI and PsbX genes for photosystem I and II, respectively) and ribosomal genes. Photosystem I and II are the two multi-protein complexes that use the light energy to catalyze the primary photosynthetic endergonic reactions producing high energy compounds^31^. Photosynthesis genes have been also found in MAGs recovered from the photo-oxic-zone in Shark Bay mats^4^.

On the other hand, layers 3 and 4 contained the greatest coverages of archaea and viruses (Fig. 1, S4, S6). For archaea domain, the phyla Euryarchaeota, and the family *Methanosarcinaceae*, were the dominant taxa. Some archaea are known to live in extreme environments such as high salt or temperature due to having diverse energy sources, cell physiology and metabolic characteristics. Previous studies in GN found Euryarchaeota and *Methanosarcinaceae* as predominant methylotrophic methanogenesis archaea using 16S rRNA amplicon sequencing^32^. Moreover, we also identified an uncultured archaeal MSBL1, described as a lineage exclusive to hypersaline environments^33^. Archaeal well-known to participate in the cycle of nitrogen, sulfur, methane and carbon in other hypersaline microbial mat from Shark Bay, Australia^34^ were also detected in the GN microbial mat. Archaeal classified genes related to nitrogen, sulfur, methane and carbon cycles were detected and distributed across multiple microbial taxa (Table S20). Assimilatory pathway of nitrogen fixation genes were annotated in genes classified as originating from Candidatus Micrarchaeota and Euryarchaeota, while nitrate reductase gamma subunit, nitric oxide reductase NorD protein, anaerobic nitric oxide reductase flavorubredoxin, nitronate monooxygenase and nitrous oxidase accessory protein were present in the archaea lineages Euryarchaeota, Candidatus Thorarchaeota and Candidatus Helarchaeota (Table S20). Sulfur metabolism were identified with genes involved in dissimilatory sulfate reduction and assimilatory sulfate reduction pathways. Moreover, were also found genes to encoded polysulfide metabolism, thiosulfate reduction and thiosulfate oxidation. For methane cycle, CO_2_ to methane pathway were detected with *fwdE, fmdE* genes. With respect to carbon cycle, were identified genes involved in Arnon–Buchanan cycle, Calvin–Benson cycle and Wood–Ljungdahl pathway.

The spatial distribution of bacteria, archaea and eukarya in Guerrero Negro microbial mat has been previously surveyed to some extent, largely based on marker genes^35^; however, to the best of our knowledge no studies investigating viruses in GN have been reported. Layers 3 and 4 also contained the highest coverages of genes and the most abundant pathways for viral-classified genes were related to the recombination of proteins. A pathway related to oxidative damage repair was detected in Layer 2 with a gene related to UV damage present in uncultured *Caudoviricetes* phage. One gene was annotated as chaperonin GroES and was present in *Prokaryotic* dsDNA virus sp. (Table S18; ID 521570). Chaperonins are ancestral proteins detected in all cellular life, however, they have only been reported in a few viruses. GroES belongs to chaperonin Group I, which are involved in phage assembly^36^. The majority of the viral-classified genes (94.4%) were not successfully annotated by our KO-annotation process, highlighting the general underrepresentation of viruses in standard databases, as well as the potential great virome that awaits discovery in these mats. Similar limitations in the identification of viruses have been previously reported^37^.

In terms of abundance, viruses are the most dominant biological entities on Earth^38^. Viruses are also distributed in extreme environment of temperature, pH, salinity and in extreme dryness, such as hydrothermal vent, oil fields, deserts or deep in the subsurface^39^. They are also critically important in terms of their ecological implications in the transmission of genetic information, the predation of prokaryotic communities, and effects on nutrient cycling^40^. In this study, *Caudoviricetes* was the dominant class, comprising bacterial and archaeal viruses with head tall morphology such as podovirus, myovirus and siphovirus. The class *Caudoviricetes* is one of the most predominant viruses and its functional capabilities are relatively well characterized^41^. In other hypersaline microbial mats and in other extreme environment such as stratified sulfidic mine tailings, podovirus, myovirus and siphovirus were also the dominant viruses discovered using metagenomic sequencing^42–45^. In the current study, 67 of 149 species of viruses identified were related to known phages. The majority of the phages belonged to podovirus, myovirus and siphovirus and the families *Herelleviridae* and *Autographiviridae*, all members of the class *Caudoviricetes*. In environmental samples, 1 to 10 phages per prokaryote cell have been reported^46^. Bacteriophages specifically from podovirus, myovirus and siphovirus have been previously described in another microbial mat^43,47^. Seven species belonged to myovirus: *Cafeteria roenbergensis* virus, *Barrevirus* sp., *Homavirus* sp., *Edafosvirus* sp., *Klosneuvirus* KNV1, *Mimivirus* LCMiAC01 and *Catovirus* CTV1. Myovirus compresses a nucleo-cytoplasmic large DNA viruses (NCLDV) called “giant viruses” that infect eukaryotic cells^48,49^. We also detected a gene taxonomically classified as the virophage *Cafeteria roenbergensis*, which increased with depth (Table S18; ID 302828), that was not successfully functionally annotated. *Cafeteria roenbergensis* is a double-stranded DNA virus, reported in the infection of marine microflagellates and contains a genome around 730 kb^50^.

In this study, genes potentially related to adaptation to the extreme living conditions were detected, specifically functions related to UV radiation resistance, multidrug resistance, oxidative stress, heavy metal, antioxidative stress, salinity and desiccation stress conditions. This included 6477 bacterial and 44 archaeal genes (Fig. 2A, B, Tables S10–S17). Furthermore, one gene related to UV DNA damage endonuclease that was taxonomically classified as viral was detected (Fig. 2C, Table S18). Genes involved with responses to oxidative stress, metal toxicity, osmotic stress due to hypersalinity, high-UV irradiation or protection against viral damage have been reported in Shark Bay microbial mats in Australia via metagenomics analysis^4^. Wong study^4^ is consistent with other studies^51–53^ in which genes related to adaptation are potentially more abundant under extreme conditions. In extreme environments like brine, Antarctic lakes, or permafrost, genes related to metal resistance, cold shock, multidrug resistance efflux pumps, and resistance to antibiotic stress conditions have been identified in greater frequencies relative to other environments^51^.

Microbial mats have existed for around 85% of the Earth’s history, reaching back prior to the emergence of oxygenic photosynthesis and 997 UV-related genes were classified under the domain Bacteria, with 14 under the archaeal domain (Tables S15 and S17). It is tempting to speculate that UV-related genes recovered from these mats may provide insight into this evolutionary history. Before oxygenic photosynthesis, no protective ozone layer was present and microorganisms may have experienced a greater selective pressure to develop strategies to protect themselves against ultraviolet and gamma irradiation than they do on the modern Earth. Moreover, the presence of UV resistant genes in the bottom layer might be related to microbial metabolism and the reduction of the damage produced by photo-oxidative products or DNA protection or damage repair. Layers 3 and 4 are mostly anoxygenic and generally dominated by anaerobic or mixotrophic lifestyles. For bacteria, *Proteobacteria*, *Chloroflexi*, *Bacteriodetes* and *Planctomycetes* were prominent phyla identified with UV genes. For Archaea, we found *Euryarchaeota* and *Methanosarcinaceae* as predominant methylotrophic methanogens, and moreover, coverages increased in bottom layers. Additionally, UV resistant genes were detected from *Euryarchaeota*, Candidatus *Bathyarchaeota*, Candidatus *Korarchaeota* and Candidatus *Thorarchaeota* archaeal lineages. In mats located in Shark Bay, Australia, high-UV exposure has been correlated with low rainfall, high salinity, high evaporation and variations in the extent of the ozone layer in hypersaline microbial mat^54^. Additionally, photoprotective strategies against UV radiation or reactive oxygen species have been identified in other cyanobacteria-dominated microbial mats^55^. As commented above, microbial mats are one of the most ancient ecosystems known and arsenic metabolism was an energetic metabolism in the primitive Earth^56^. In the metagenome, genes encoding arsenical resistance were affiliated to bacteria lineages of Cyanobacteria and Proteobacteria (Table S11). Arsenical resistance genes has been detected in modern stromatolites in Andean microbial ecosystems in Argentina and in Shark Bay microbial mats in Australia using metagenomics^4,56^.

Guerrero Negro mats are found in extreme salinity, and large diel fluctuations of oxygen, hydrogen sulfide, pH, CO_2_ and nutrients. This ecosystem is poor in nutrients, and the presence of multidrug resistance genes and antibiotics may offer a competitive advantage^57,58^. Multidrug-resistance genes were detected with 930 genes in bacteria and 6 genes in Archaea. Multidrug-resistance genes have also been noted in Antarctic microbial mats^59^. The role of multidrug-resistance genes in mats is not yet clear however, and might be correlated with a general increased potential for adaption adaptation of microbes living in relatively extreme conditions as has been suggested in a recent study^51^. In addition, as a result of diel cycle (24 h) fluctuations of oxygen in the mat, genes annotated as superoxide-dismutase, superoxide oxidase and superoxide reductase might help to protect anaerobic microorganisms from oxidative stress^4,60^. Genes encoding the oxidative stress genes were found in bacteria and in archaea lineages (Tables S13 and S17). In bacteria, superoxide dismutase FeMn (*SOD2*) were the most abundant (highest coverage) of the oxidative stress genes and were more abundant in the upper layers where oxygen concentration is elevated (Fig. 2A). Heat and cold shock proteins were also found, and may reflect the stress induced by diurnal-seasonal cycles along environmental temperature changes. Cold-shock protein was the annotation for 221 genes in bacteria and 7 genes in archaea and was more abundant in Layers 3 and 4 (Fig. 2A, B; Tables S14 and S17). Cold-shock protein (*Csp*) is well characterized in bacteria; however, it has been reported that most archaeal genomes do not contain *csp* genes^61^ or at least do not contain detected homologs to the bacterial *csp*^62^. In this study, *cspA* gene was present in 3 archaeal phyla: Candidatus Aenigmarchaeota, Euryarchaeota and Candidatus Woesearchaeota (Table S17). In addition to cold stress protection, bacterial *csps* offer other biological functions such as antioxidative stress, anti-antibiotic activity and stationary growth^63^. In GN, cold-shock protein may play a protective role in the response to evolution of environmental conditions such as temperature downward change, oxidative stress or variations of pH, offering cells protection against the news conditions. With regard to heat shock proteins (*hsIJ*), 184 genes were present in bacteria and 7 in archaea (Tables S14 and S17). In addition to preventing the thermal denaturing of proteins, heat shock proteins may be also associated with cold and UV stress^64–66^.

Furthermore, living in low water availability, microorganism have strategies to balance their internal osmolarity by accumulation those low molecular solutes (osmoprotectants)^67^. In this study, were identified intracellular reserves as trehalose, glycine-betaine, glutamate or ectoine solutes (called osmoprotectants). In the metagenome were detected genes associated with high desiccation and salinity conditions, such as involved in the production of exopolysaccharides, Na+-transporting mechanism, molecular chaperones and osmoprotectants. The greatest coverages were detected for genes involved with exopolysaccharides (*rpoE*), F-type H+/Na+-transporting ATPase subunit beta (*ATPF1B, atpD*), and molecular chaperones (*GroES, GroEL, DnaJ* and *DnaK*). Molecular chaperones are shock response genes under xeric stress, *rpoE* gene play a role in the production of exopolysaccharides and is involved in the control of misfolding proteins; and *ATPF1B, atpD* gene is related with Na+-transporting mechanism for prevent excessive sodium ion against high salinity^67,68^.

Guerrero Negro microbial mat reside in dense and metabolically interdependent communities, comprising microorganisms from all lineages of life (Bacteria, Eukarya, Archaea and viruses). In microbial mats, has been documented microbial interactions between different functional microorganism such as Cyanobacteria, sulfate reducers/sulfate oxidizers and methanogens^71–73^. We employed phylogenetics in order to investigate the evolutionary histories of genes with similar functional annotations that were taxonomically classified as coming from bacteria, archaea and viruses (Table 1). Phylogenetic analyses in Figs. 3, 4, 5 and 6 and S7–S15 showed an interlinking between domains, where genes taxonomically classified as archaea and viruses formed clades. A total of 12 genes and 1779 sequences related to potential mechanisms of adaptation, were analyzed. The genes were present in bacteria, archaea and viruses, with 1743, 35 and 1 sequence, for bacteria, archaea and viruses, respectively. In all the trees built, the genes present in archaea were phylogenetically relatively closely related to genes present in bacteria lineages—suggesting they might have been acquired from the bacterial domain. Genes present in archaea lineages of Euryarchaeota (Candidatus *Methanolliviera hydrocarbonicum*, *Archaeoglobales* archaeon, *Methanothermobacter*), Candidatus Micrarchaeota, Candidatus Thermoplasmatota (*Thermoplasmatales*, *Thermoplasmata, Methanomassiliicoccalesa*), Candidatus Aenigmarchaeota (Candidatus *Aenigmarchaeota* archaeon), and Candidatus Woesearchaeota were closely related with bacterial lineages of Actinomycetota, Bacillota (*Peptoclostridium littorale*, *Clostridia*), Bacteroidota (Bacteroidetes *bacterium*, *Tangfeifania diversioriginum*), Candidatus Pacebacteria (Candidatus Pacebacteria *bacterium*), Chloroflexota (Chloroflexi *bacterium*), candidate division Zixibacteria, candidate division KSB1, Candidatus Cloacimonetes (Candidatus *Cloacimonas* sp.), Pseudomonadota (*Desulfonemais himotonii*, Proteobacteria *bacterium*, *Desulfobacterales*), Spirochaetota (Spirochaetes *bacterium*, Spirochaetaceae *bacterium*) and Verrucomicrobiota (*Puniceicoccus vermicola*). High salinity may favorable the gene gain between life-forms in the mat since create a mix conditions of electron acceptors and electron donors, nutrients, and carbon sources in the ionic and redox gradient, as has been reported^74^. Besides, one *uvsE*,* UVE1* gene present in uncultured *Caudoviricetes* virus formed a clade with genes presents in bacteria and archaea lineages. Viruses can be vehicles for horizontal gene transfer (HGT) between life-forms, influencing the cellular evolution in the environment^39^. In a recent study, sulfate reduction genes detected in viruses were identified as being phylogenetically closely related to bacterial lineages, suggesting they too may have been acquired from bacteria^45^. For microorganisms living in extreme environments, particularly in dense communities like microbial mats, horizontal gene transfer (HGT) is suspected to be the prominent method of gene gain in bacteria and archaea^69,70^. Further studies are needed in order to elucidate the interlinking mechanism between genes present in the different domains in GN.

**Table 1:** Phylogenetically investigated KO functions.

KO definition	KO ID	Gene name(s)	Bacteria	Archaea	Virus	Figures/Tables
MFS transporter, ACDE family, multidrug resistance protein	K08221	yitG, ymfD, yfmO	14	1	0	Figure 3A, Tables S10, S17
Small multidrug resistance pump	K03297	emrE, qac, mmr, smr	21	1	0	Figure 3B, Tables S10, S17
Superoxide dismutase, Fe–Mn family	K04564	SOD2	171	1	0	Figure 4A, S7, Tables S13, S17
Superoxide reductase	K05919	dfx	107	7	0	Figure 4B, S8, Tables S13, S17
Heat shock protein HtpX	K03799	htpX	106	4	0	Figure 5A, S9, Tables S14, S17
Cold shock protein	K03704	cspA	221	7	0	Figure 5B, S10, Tables S14, S17
Phage shock protein C	K03973	pspC	114	2	0	Figure 5C, S11, Tables S14, S17
Excinuclease ABC subunit A	K03701	uvrA	362	5	0	Figure 6A, S12, Tables S15, S17
Excinuclease ABC subunit B	K03702	uvrB	193	1	0	Figure S13, Tables S15, S17
Excinuclease ABC subunit C	K03703	uvrC	203	3	0	Figure 6B, S14, Tables S15, S17
DNA helicase II ATP dependent DNA helicase	K03657	uvrD, pcrA	214	1	0	Figure S15, Tables S15, S17
UV DNA damage endonuclease	K13281	uvsE, UVE1	17	2	1	Figure 6C, Tables S15, S17, S18

## Conclusion

In this study, a community-wide gene-focused analysis showed a vertical partitioning of bacteria, archaea, eukarya and viruses and their functional potential. According to unique genes recovered, the mat was found to be composed by bacteria (98.01%), archaea (1.81%), eukarya (0.07%) and virus (0.11%). The greatest coverages of genes of bacteria and eukarya were detected in the upper layers, while the highest coverages of genes of archaea and viruses were found in deeper layers. Many genes related to UV radiation, multidrug resistance, oxidative stress, heavy metals, antioxidative stress, salinity, and desiccation were detected. Genes such as these were taxonomically classified as belonging to bacteria, archaea and viruses (with 6477, 44 and 1 genes, respectively). Phylogenetic analysis revealed a potential interlinkage between genes present in bacteria, archaea, and viruses. To sum up, this study makes contributions to the understanding of the adaptation mechanism in an extreme environment and how microbes can adapt to their environment, providing new light regard to interlinking between lifeforms in GN mat.

### Supplementary Information

Supplementary Tables.

Supplementary Figures.

## Data Availability

The datasets generated and analysed during the current study are available in the National Center for Biotechnology Information repository, Accession: PRJNA688760.
