# Supplementary material for: Community ecology and functional potential of bacteria, archaea, eukarya and viruses in Guerrero Negro microbial mat
Source: Sci Rep. 2024 Jan 31;14:2561. doi: 10.1038/s41598-024-52626-y (PMC10831059; doi:10.1038/s41598-024-52626-y)
Supplement: Supplementary file 2 — Supplementary Figures. [file 41598_2024_52626_MOESM2_ESM.pdf]

Supplementary Material

Community ecology and functional potential of bacteria, archaea, eukarya and viruses in Guerrero Negro microbial mat

P. Maza-Márquez[1,2], M.D. Lee[1,3], B.M. Bebout[3]

[1]Exobiology Branch, NASA Ames Research Center, Moffett Field, CA, USA.
[2]University of Granada, Spain.
[3]Blue Marble Space Institute of Science, Seattle, WA, USA.

***Correspondence:**

Paula Maza-Márquez

paulamazamar@gmail.com

Supplementary Figures S1-S15

Supplementary Figure 1. Location of the study sited in Guerrero Negro, Baja California, Mexico. Figure from P Maza-Márquez, M D Lee, A M Detweiler, B M Bebout: Millimeter-scale vertical partitioning of nitrogen cycling in hypersaline mats reveals prominence of genes encoding multi-heme and prismane proteins, The ISME Journal, Volume 16, Issue 4, April 2022, Page 1202, https://doi.org/10.1038/s41396-021-01180-w

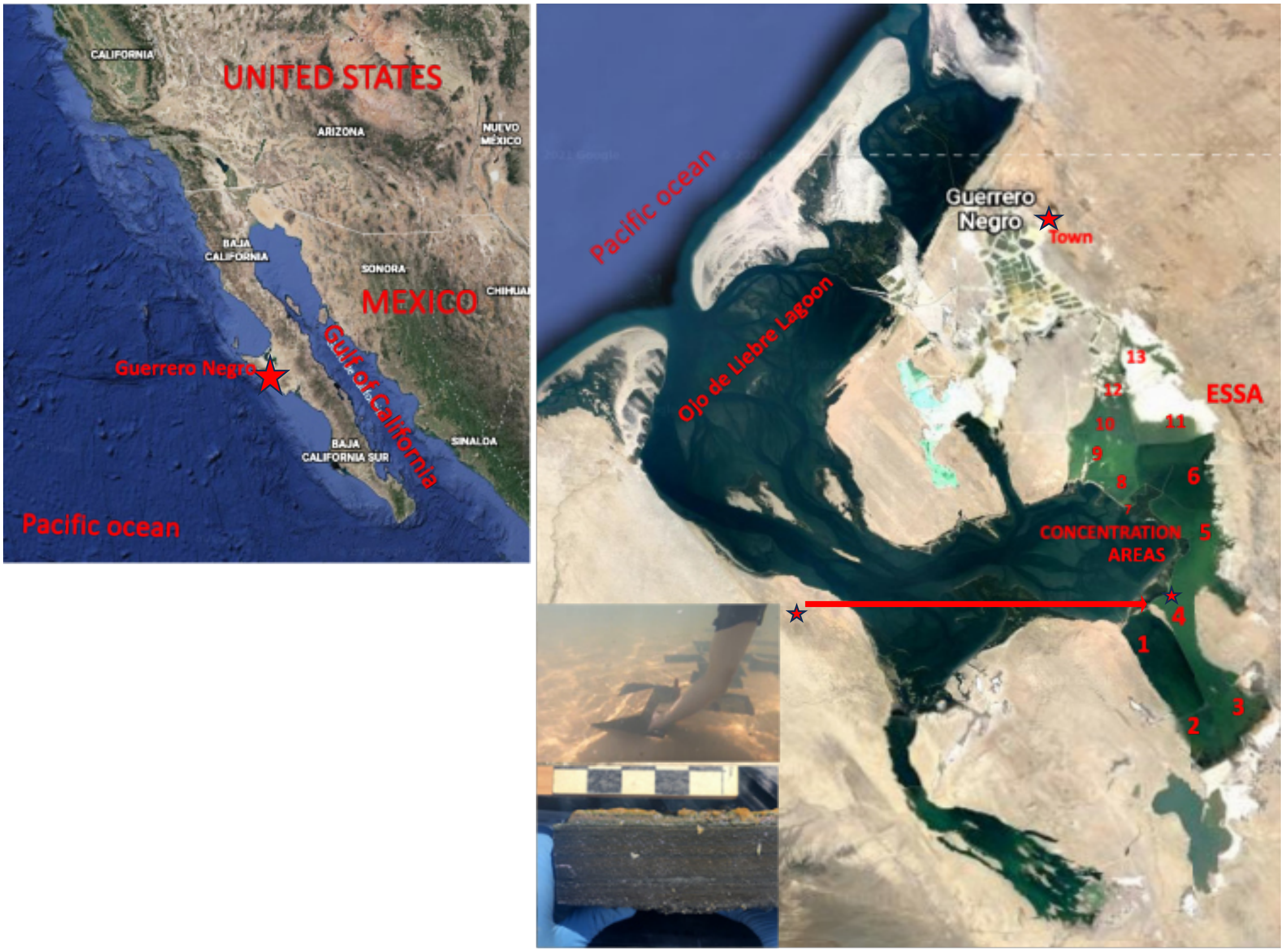

Supplementary Figure 2. **(A1)** Heatmap and cluster analysis showing the similarity of genes with >= 9 CPM in bacteria across the uppers 4 layers examined. (**A2)** Heatmap and cluster analysis showing the similarity of 14148 genes of archaea across the uppers 4 layers examined. (**A3)** Heatmap and cluster analysis showing the similarity of 547 genes of eucaryotes across the uppers 4 layers examined. (**A4)** Heatmap and cluster analysis showing the similarity of 835 genes of viruses across the uppers 4 layers examined.

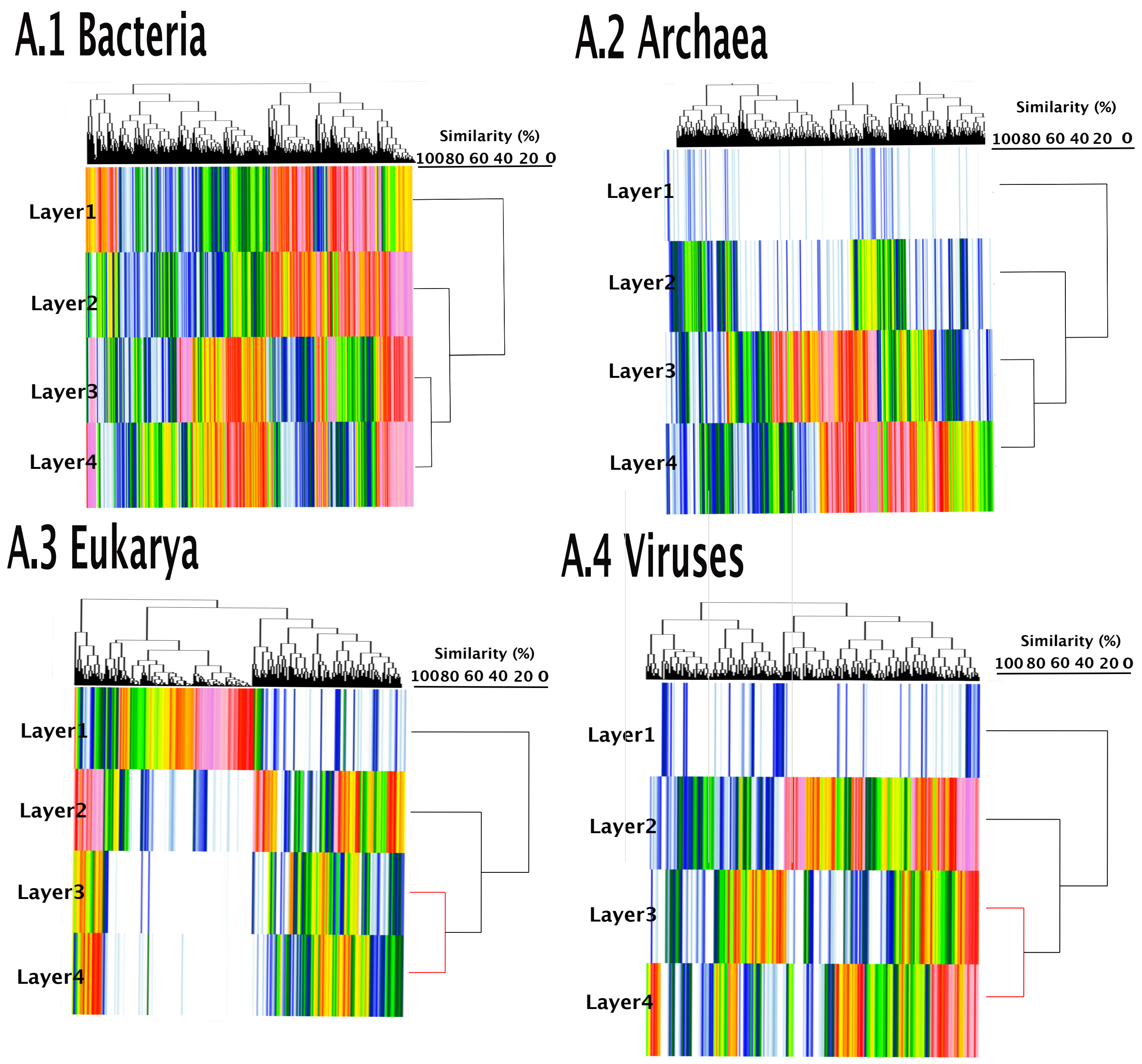

Supplementary Figure 3. Bar plots show the read-based taxonomic classification of bacteria at Phylum, and Family in the upper 4 layers examined.

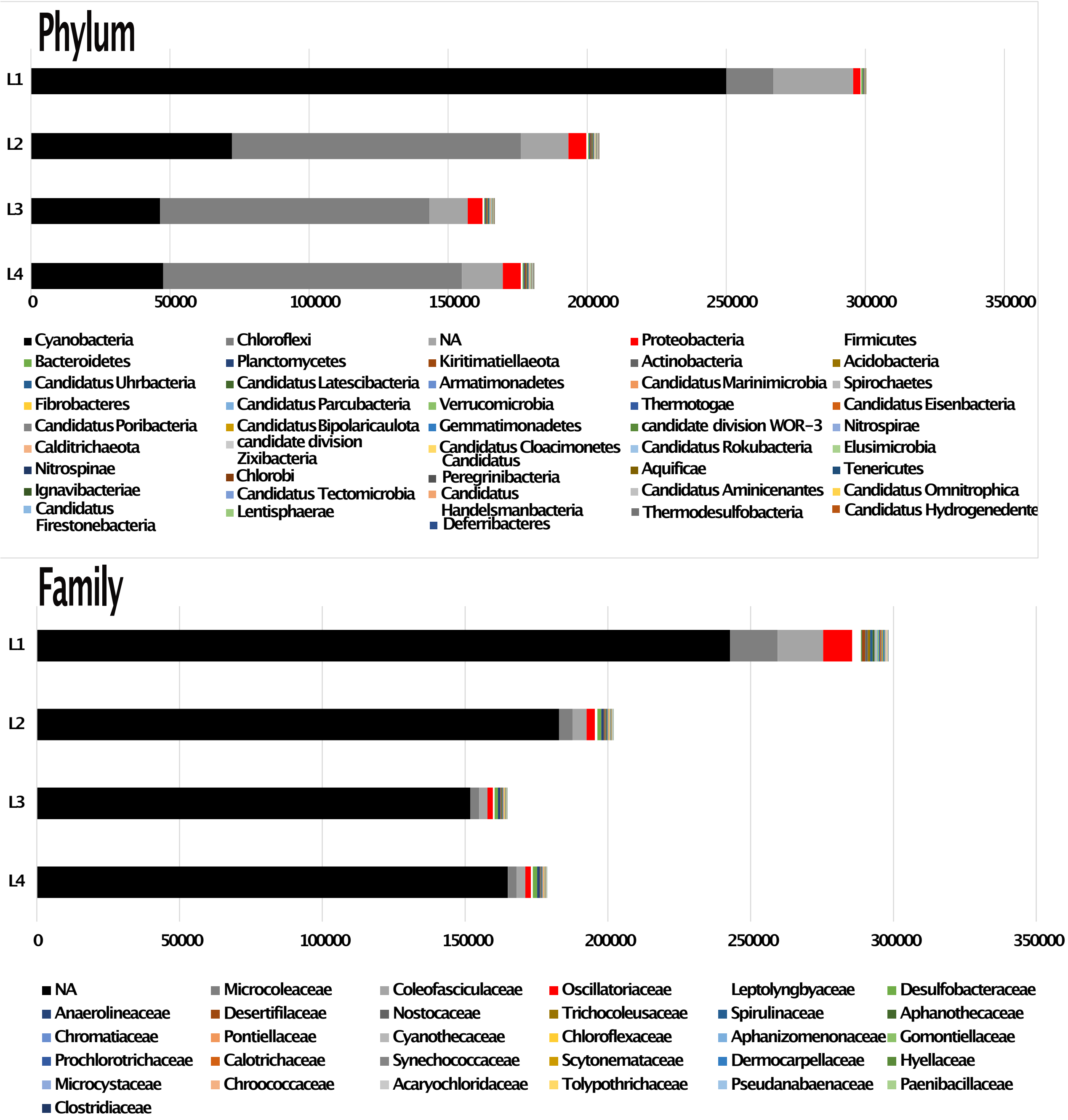

Supplementary Figure 4. Bar plots show the read-based taxonomic classification of archaea at Phylum and Family in the upper 4 layers examined.

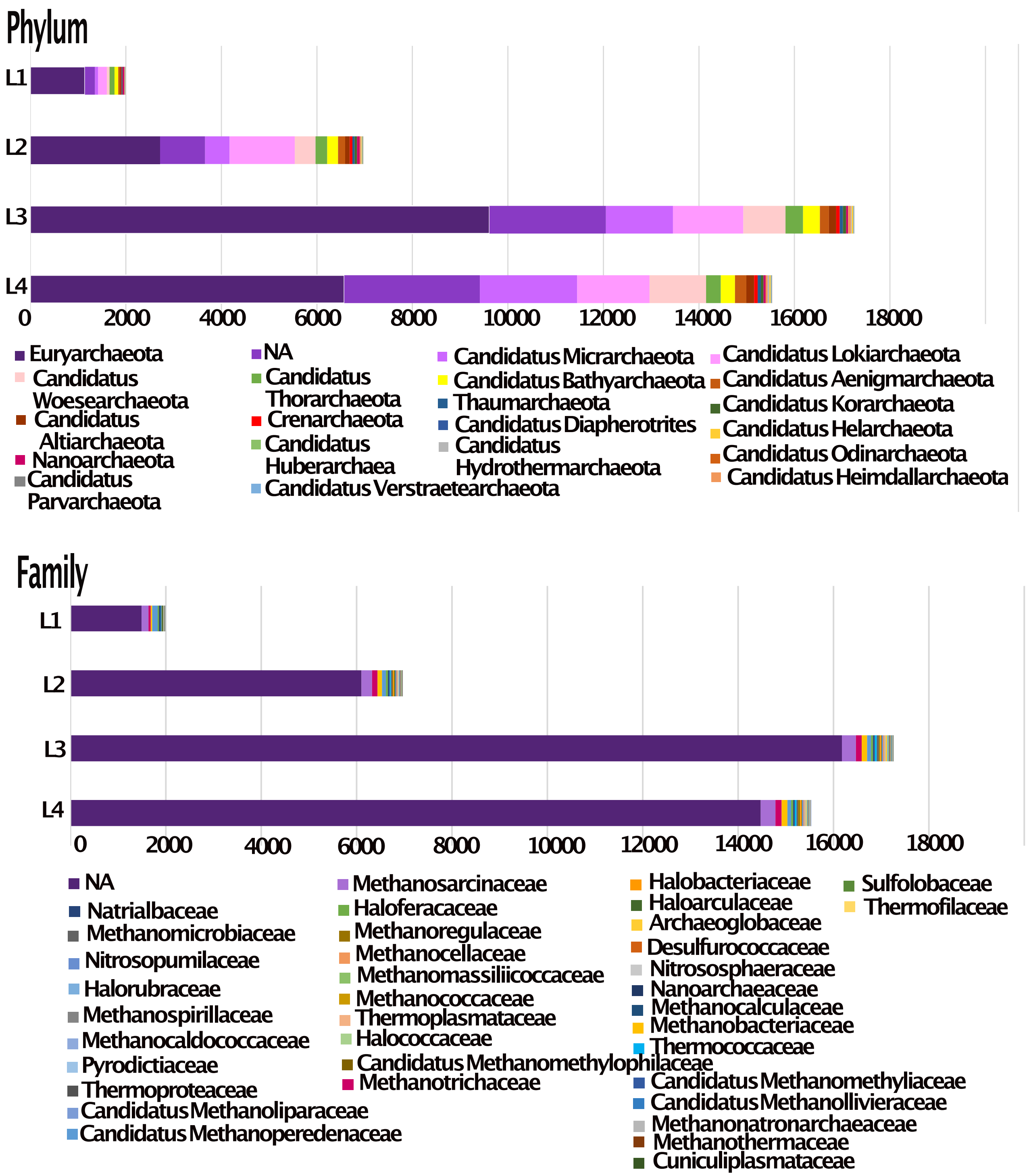

Supplementary Figure 5. Bar plots show the read-based taxonomic classification of eukarya at Phylum and Family in the uppers 4 layers examined.

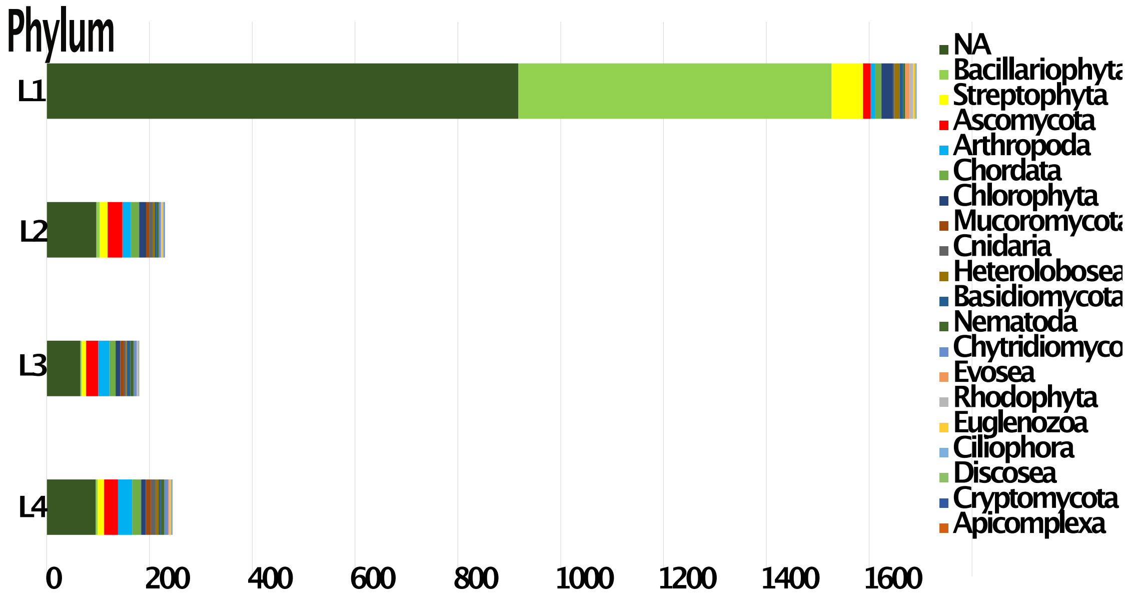

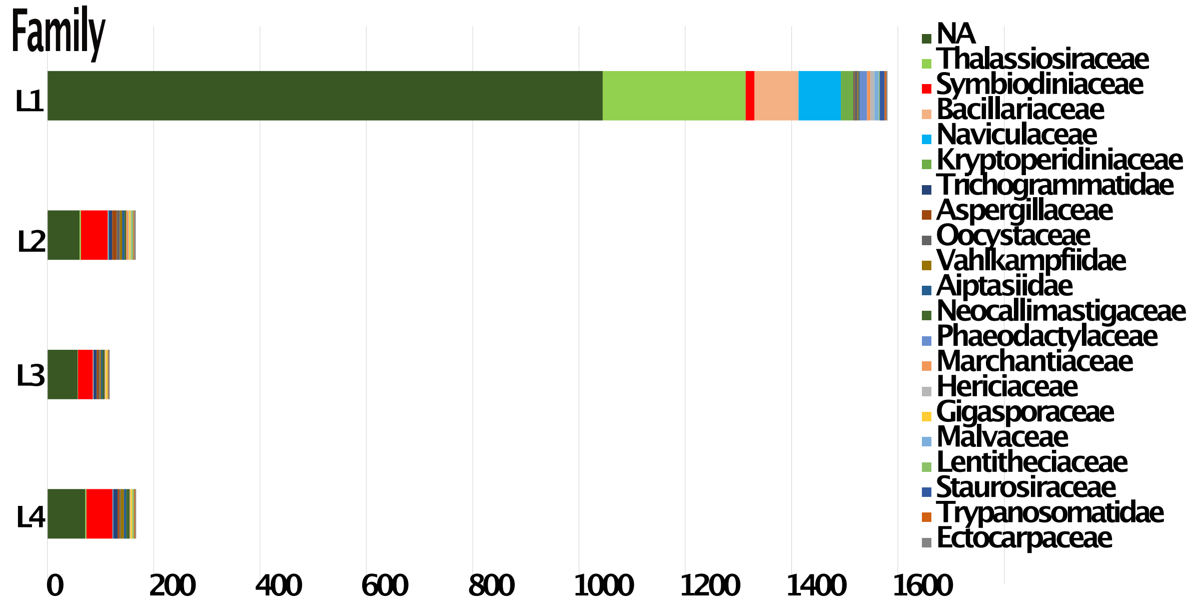

Supplementary Figure 6. Bar plots show the read-based taxonomic classification of viruses at Phylum and Family in the upper 4 layers examined.

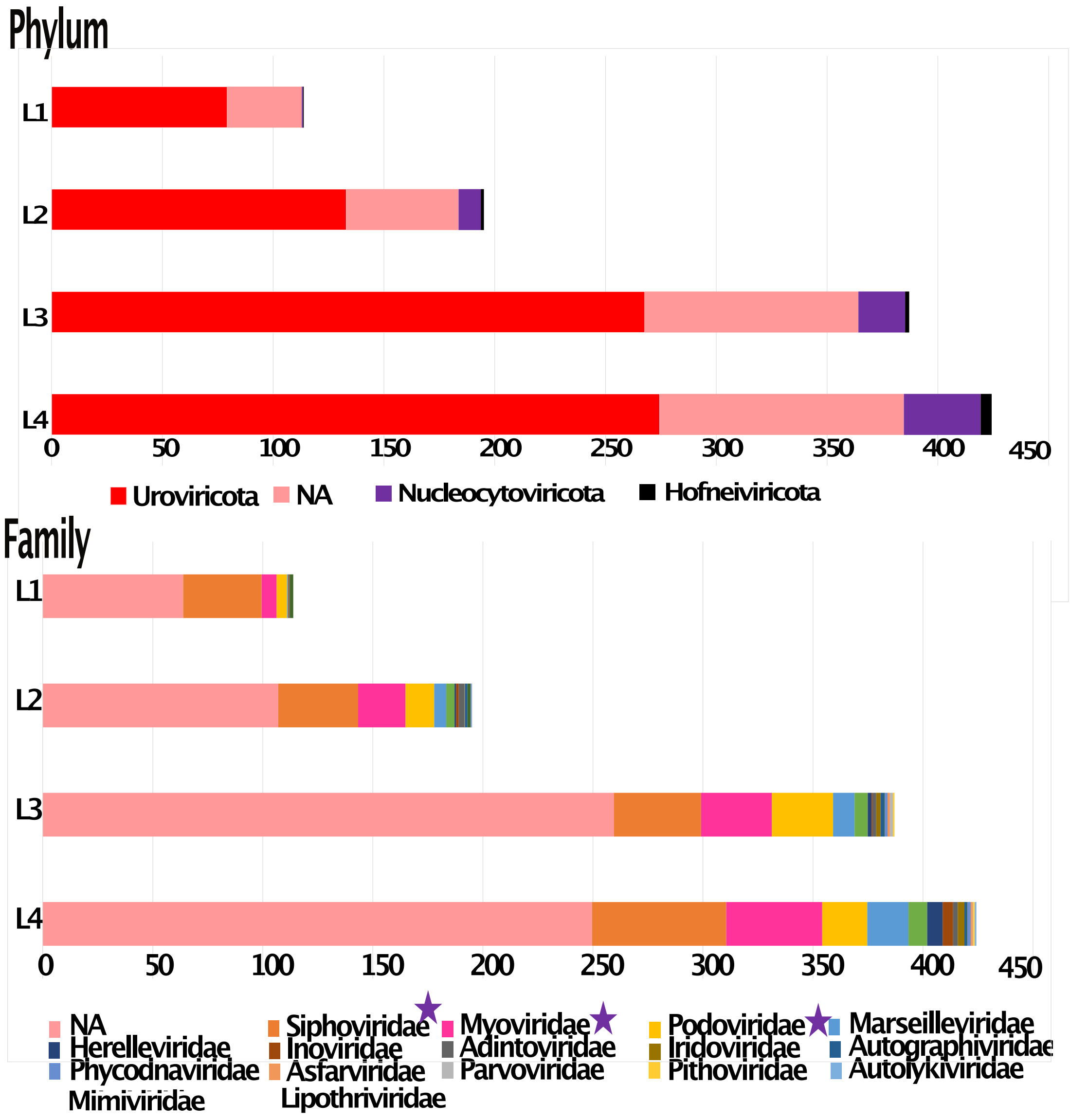

★ *Myoviridae*, *Podoviridae* and *Siphoviridae* families are recommended to be abolished in the new virus taxonomy

Supplementary Figure 7. Phylogenetic tree based on amino-acid sequences of superoxide dismutase, Fe-Mn family (*SOD2*).

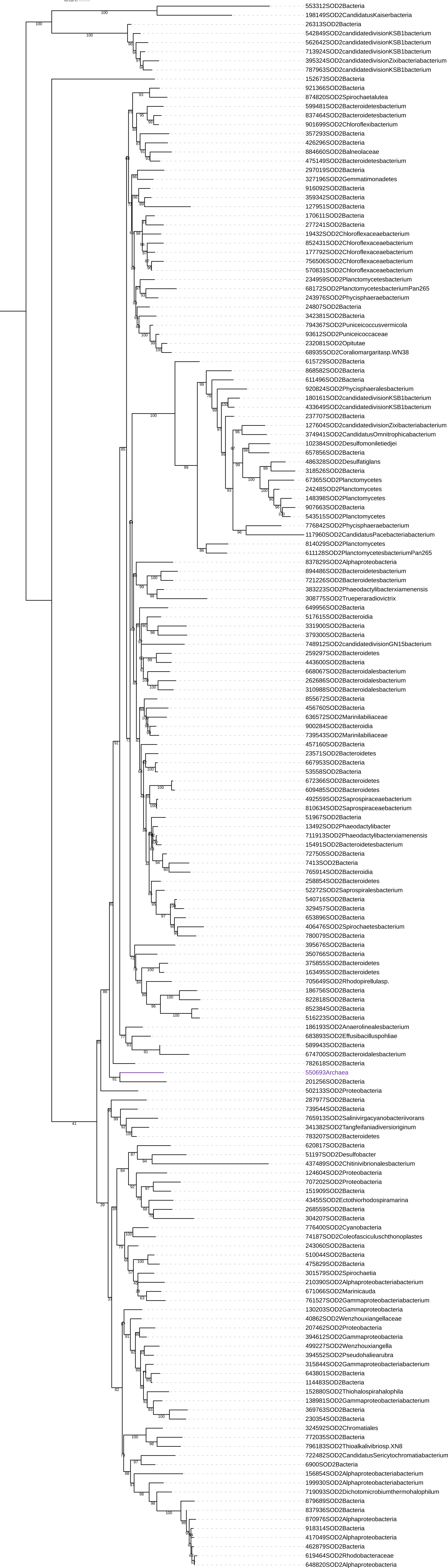

553312SOD2Bacteria
198149SOD2CandidatusKaiserbacteria
26313SOD2Bacteria
542849SOD2candidatedivisionKSB1bacterium
562642SOD2candidatedivisionKSB1bacterium
713924SOD2candidatedivisionKSB1bacterium
395324SOD2candidatedivisionZixibacteriabacterium
787963SOD2candidatedivisionKSB1bacterium
152673SOD2Bacteria
921366SOD2Bacteria
874820SOD2Spirochaetalutea
599481SOD2Bacteroidetesbacterium
837464SOD2Bacteroidetesbacterium
901699SOD2Chloroflexibacterium
357293SOD2Bacteria
426296SOD2Bacteria
884660SOD2Balneolaceae
475149SOD2Bacteroidetesbacterium
297019SOD2Bacteria
327196SOD2Gemmatimonadetes
916092SOD2Bacteria
359342SOD2Bacteria
127951SOD2Bacteria
170611SOD2Bacteria
277241SOD2Bacteria
19432SOD2Chloroflexaceaebacterium
852431SOD2Chloroflexaceaebacterium
177792SOD2Chloroflexaceaebacterium
756506SOD2Chloroflexaceaebacterium
570831SOD2Chloroflexaceaebacterium
234959SOD2Planctomycetesbacterium
68172SOD2PlanctomycetesbacteriumPan265
243976SOD2Phycisphaeraebacterium
24807SOD2Bacteria
342381SOD2Bacteria
794367SOD2Puniceicoccusvermicola
93612SOD2Puniceicoccaceae
232081SOD2Opitutae
68935SOD2Coraliomargaritasp.WN38
615729SOD2Bacteria
868582SOD2Bacteria
611496SOD2Bacteria
920824SOD2Phycisphaeralesbacterium
180161SOD2candidatedivisionKSB1bacterium
433649SOD2candidatedivisionKSB1bacterium
237707SOD2Bacteria
127604SOD2candidatedivisionZixibacteriabacterium
374941SOD2CandidatusOmnitrophicabacterium
102384SOD2Desulfomonilетiedjei
657856SOD2Bacteria
486328SOD2Desulfatiglans
318526SOD2Bacteria
67365SOD2Planctomycetes
24248SOD2Planctomycetes
148398SOD2Planctomycetes
907663SOD2Bacteria
543515SOD2Planctomycetes
776842SOD2Phycisphaeraebacterium
117960SOD2CandidatusPacebacteriabacterium
814029SOD2Planctomycetes
611128SOD2PlanctomycetesbacteriumPan265
837829SOD2Alphaproteobacteria
894486SOD2Bacteroidetesbacterium
721226SOD2Bacteroidetesbacterium
383223SOD2Phaeodactylibacterxiamenensis
308775SOD2Trueperaradiovictrix
649956SOD2Bacteria
517615SOD2Bacteroidia
331900SOD2Bacteria
379300SOD2Bacteria
748912SOD2candidatedivisionGN15bacterium
259297SOD2Bacteroidetes
443600SOD2Bacteria
668067SOD2Bacteroidalesbacterium
262686SOD2Bacteroidalesbacterium
310988SOD2Bacteroidalesbacterium
855672SOD2Bacteria
456760SOD2Bacteria
636572SOD2Marinilabiliaceae
900284SOD2Bacteroidia
739543SOD2Marinilabiliaceae
457160SOD2Bacteria
23571SOD2Bacteroidetes
667953SOD2Bacteria
53558SOD2Bacteria
672366SOD2Bacteroidetes
609485SOD2Bacteroidetes
492559SOD2Saprospiraceaebacterium
810634SOD2Saprospiraceaebacterium
51967SOD2Bacteria
13492SOD2Phaeodactylibacter
711913SOD2Phaeodactylibacterxiamenensis
15491SOD2Bacteroidetesbacterium
727505SOD2Bacteria
7413SOD2Bacteria
765914SOD2Bacteroidia
258854SOD2Bacteroidetes
52272SOD2Saprospiralesbacterium
540716SOD2Bacteria
329457SOD2Bacteria
653896SOD2Bacteria
406476SOD2Spirochaetesbacterium
780079SOD2Bacteria
395676SOD2Bacteria
350766SOD2Bacteria
375855SOD2Bacteroidetes
163495SOD2Bacteroidetes
705649SOD2Rhodopirellulasp.
186756SOD2Bacteria
822818SOD2Bacteria
852384SOD2Bacteria
516223SOD2Bacteria
186193SOD2Anaerolinealesbacterium
683893SOD2Effusibacilluspohliae
589943SOD2Bacteria
674700SOD2Bacteroidalesbacterium
782618SOD2Bacteria
550693Archaea
201256SOD2Bacteria
502133SOD2Proteobacteria
287977SOD2Bacteria
739544SOD2Bacteria
765913SOD2Salinivirgacyanobacteriivorans
341382SOD2Tangfeifaniadiversioriginum
783207SOD2Bacteroidetes
620817SOD2Bacteria
51197SOD2Desulfobacter
437489SOD2Chitinivibrionalesbacterium
124604SOD2Proteobacteria
707202SOD2Proteobacteria
151909SOD2Bacteria
43455SOD2Ectothiorhodospiramarina
268559SOD2Bacteria
304207SOD2Bacteria
776400SOD2Cyanobacteria
74187SOD2Coleofasciculuschthonoplastes
243060SOD2Bacteria
510044SOD2Bacteria
475829SOD2Bacteria
301579SOD2Spirochaetia
210390SOD2Alphaproteobacteriabacterium
671066SOD2Marinicauda
761527SOD2Gammaproteobacteriabacterium
130203SOD2Gammaproteobacteria
40862SOD2Wenzhouxiangellaceae
207462SOD2Proteobacteria
394612SOD2Gammaproteobacteria
499227SOD2Wenzhouxiangella
394552SOD2Pseudohaliearubra
315844SOD2Gammaproteobacteriabacterium
643801SOD2Bacteria
114483SOD2Bacteria
152880SOD2Thiohalospirahalophila
138981SOD2Gammaproteobacteriabacterium
369763SOD2Bacteria
230354SOD2Bacteria
324592SOD2Chromatiales
772035SOD2Bacteria
796183SOD2Thioalkalivibriosp.XN8
722482SOD2CandidatusSericytochromatiabacterium
6900SOD2Bacteria
156854SOD2Alphaproteobacteriabacterium
199930SOD2Alphaproteobacteriabacterium
719093SOD2Dichotomicrobiumthermohalophilum
879689SOD2Bacteria
837936SOD2Bacteria
870976SOD2Alphaproteobacteria
918314SOD2Bacteria
417049SOD2Alphaproteobacteria
462879SOD2Bacteria
619464SOD2Rhodobacteraceae
648820SOD2Alphaproteobacteria

Supplementary Figure 8. Phylogenetic tree based on amino-acid sequences of superoxide reductase (*dfx*).

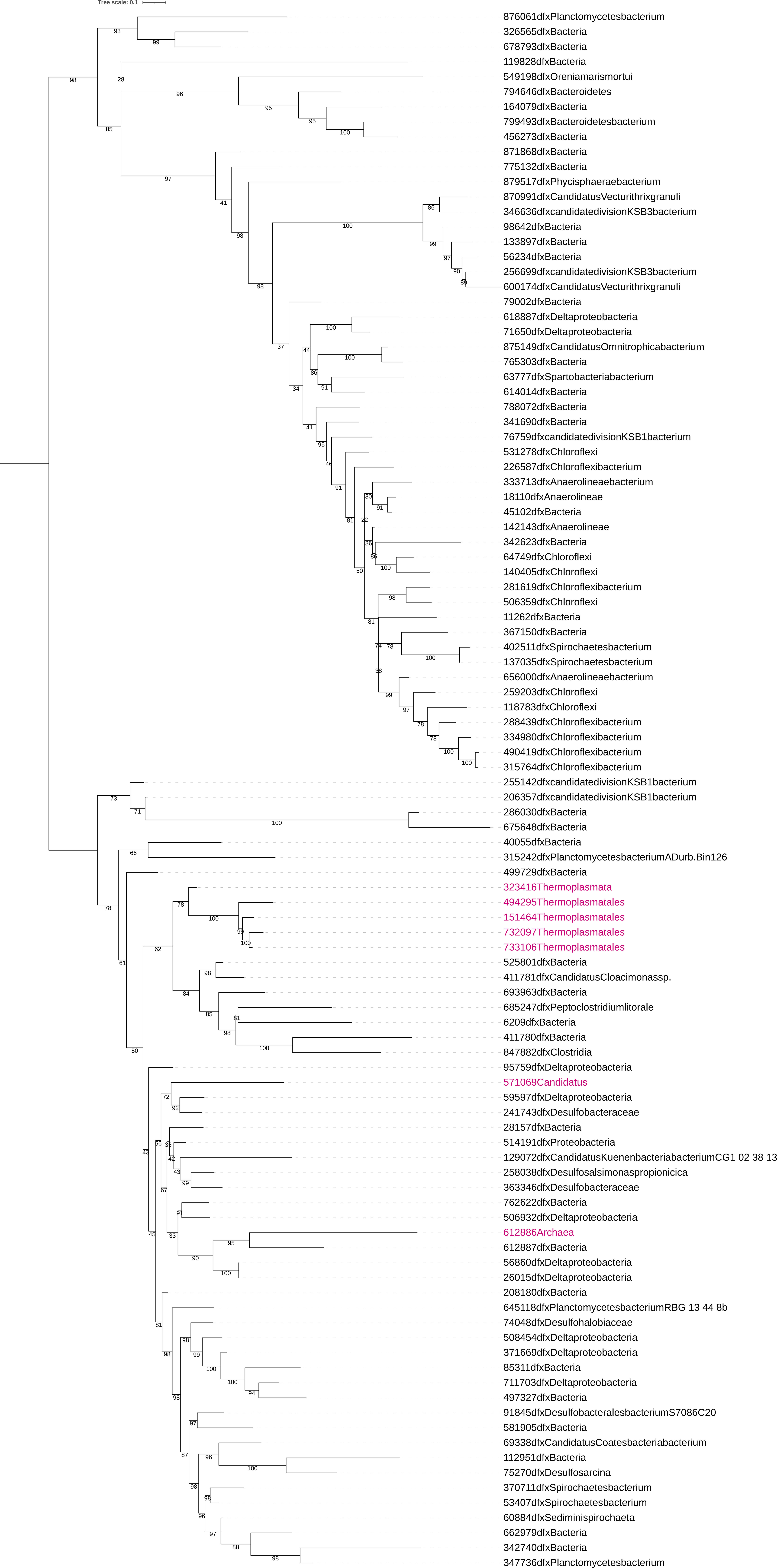
Tree scale: 0.1
876061dfxPlanctomycetesbacterium
326565dfxBacteria
678793dfxBacteria
119828dfxBacteria
549198dfxOreniamarismortui
794646dfxBacteroidetes
164079dfxBacteria
799493dfxBacteroidetesbacterium
456273dfxBacteria
871868dfxBacteria
775132dfxBacteria
879517dfxPhycisphaerae bacterium
870991dfxCandidatusVecturithrixgranuli
346636dfxcandidatedivisionKSB3bacterium
98642dfxBacteria
133897dfxBacteria
56234dfxBacteria
256699dfxcandidatedivisionKSB3bacterium
600174dfxCandidatusVecturithrixgranuli
79002dfxBacteria
618887dfxDeltaproteobacteria
71650dfxDeltaproteobacteria
875149dfxCandidatusOmnitrophicabacterium
765303dfxBacteria
63777dfxSpartobacteriabacterium
614014dfxBacteria
788072dfxBacteria
341690dfxBacteria
76759dfxcandidatedivisionKSB1bacterium
531278dfxChloroflexi
226587dfxChloroflexibacterium
333713dfxAnaerolineaebacterium
18110dfxAnaerolineae
45102dfxBacteria
142143dfxAnaerolineae
342623dfxBacteria
64749dfxChloroflexi
140405dfxChloroflexi
281619dfxChloroflexibacterium
506359dfxChloroflexi
11262dfxBacteria
367150dfxBacteria
402511dfxSpirochaetesbacterium
137035dfxSpirochaetesbacterium
656000dfxAnaerolineaebacterium
259203dfxChloroflexi
118783dfxChloroflexi
288439dfxChloroflexibacterium
334980dfxChloroflexibacterium
490419dfxChloroflexibacterium
315764dfxChloroflexibacterium
255142dfxcandidatedivisionKSB1bacterium
206357dfxcandidatedivisionKSB1bacterium
286030dfxBacteria
675648dfxBacteria
40055dfxBacteria
315242dfxPlanctomycetesbacteriumADurb.Bin126
499729dfxBacteria
323416Thermoplasmata
494295Thermoplasmatales
151464Thermoplasmatales
732097Thermoplasmatales
733106Thermoplasmatales
525801dfxBacteria
411781dfxCandidatusCloacimonassp.
693963dfxBacteria
685247dfxPeptoclostridiumlitorale
6209dfxBacteria
411780dfxBacteria
847882dfxClostridia
95759dfxDeltaproteobacteria
571069Candidatus
59597dfxDeltaproteobacteria
241743dfxDesulfobacteraceae
28157dfxBacteria
514191dfxProteobacteria
129072dfxCandidatusKuenenbacteriabacteriumCG1 02 38 13
258038dfxDesulfosalsimonaspropionicica
363346dfxDesulfobacteraceae
762622dfxBacteria
506932dfxDeltaproteobacteria
612886Archaea
612887dfxBacteria
56860dfxDeltaproteobacteria
26015dfxDeltaproteobacteria
208180dfxBacteria
645118dfxPlanctomycetesbacteriumRBG 13 44 8b
74048dfxDesulfohalobiaceae
508454dfxDeltaproteobacteria
371669dfxDeltaproteobacteria
85311dfxBacteria
711703dfxDeltaproteobacteria
497327dfxBacteria
91845dfxDesulfobacteralesbacteriumS7086C20
581905dfxBacteria
69338dfxCandidatusCoatesbacteriabacterium
112951dfxBacteria
75270dfxDesulfosarcina
370711dfxSpirochaetesbacterium
53407dfxSpirochaetesbacterium
60884dfxSediminispirochaeta
662979dfxBacteria
342740dfxBacteria
347736dfxPlanctomycetesbacterium

Supplementary Figure 9. Phylogenetic tree based on amino-acid sequences of heat shock protein HtpX (*htpX*).

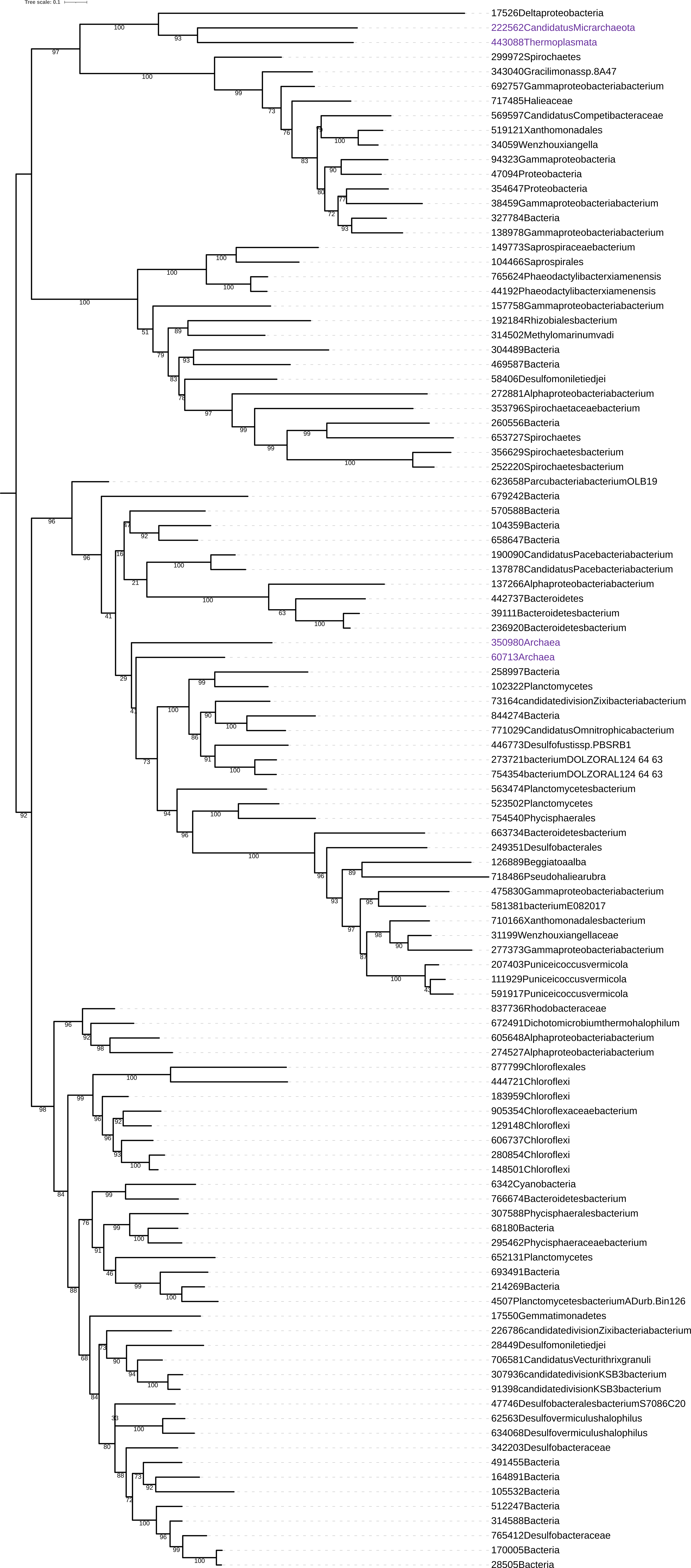
Tree scale: 0.1
17526Deltaproteobacteria
222562CandidatusMicrarchaeota
443088Thermoplasmata
299972Spirochaetes
343040Gracilimonassp.8A47
692757Gammaproteobacteriabacterium
717485Halieaceae
569597CandidatusCompetibacteraceae
519121Xanthomonadales
34059Wenzhouxiangella
94323Gammaproteobacteria
47094Proteobacteria
354647Proteobacteria
38459Gammaproteobacteriabacterium
327784Bacteria
138978Gammaproteobacteriabacterium
149773Saprospiraceaebacterium
104466Saprospirales
765624Phaeodactylibacterxiamenensis
44192Phaeodactylibacterxiamenensis
157758Gammaproteobacteriabacterium
192184Rhizobialesbacterium
314502Methylomarinumvadi
304489Bacteria
469587Bacteria
58406Desulfomoniletiedjei
272881Alphaproteobacteriabacterium
353796Spirochaetaceaebacterium
260556Bacteria
653727Spirochaetes
356629Spirochaetesbacterium
252220Spirochaetesbacterium
623658ParcubacteriabacteriumOLB19
679242Bacteria
570588Bacteria
104359Bacteria
658647Bacteria
190090CandidatusPacebacteriabacterium
137878CandidatusPacebacteriabacterium
137266Alphaproteobacteriabacterium
442737Bacteroidetes
39111Bacteroidetesbacterium
236920Bacteroidetesbacterium
350980Archaea
60713Archaea
258997Bacteria
102322Planctomycetes
73164candidatedivisionZixibacteriabacterium
844274Bacteria
771029CandidatusOmnitrophicabacterium
446773Desulfofustissp.PBSRB1
273721bacteriumDOLZORAL124 64 63
754354bacteriumDOLZORAL124 64 63
563474Planctomycetesbacterium
523502Planctomycetes
754540Phycisphaerales
663734Bacteroidetesbacterium
249351Desulfobacterales
126889Beggiatoaalba
718486Pseudohaliearubra
475830Gammaproteobacteriabacterium
581381bacteriumE082017
710166Xanthomonadalesbacterium
31199Wenzhouxiangellaceae
277373Gammaproteobacteriabacterium
207403Puniceicoccusvermicola
111929Puniceicoccusvermicola
591917Puniceicoccusvermicola
837736Rhodobacteraceae
672491Dichotomicrobiumthermohalophilum
605648Alphaproteobacteriabacterium
274527Alphaproteobacteriabacterium
877799Chloroflexales
444721Chloroflexi
183959Chloroflexi
905354Chloroflexaceaebacterium
129148Chloroflexi
606737Chloroflexi
280854Chloroflexi
148501Chloroflexi
6342Cyanobacteria
766674Bacteroidetesbacterium
307588Phycisphaeralesbacterium
68180Bacteria
295462Phycisphaeraceaebacterium
652131Planctomycetes
693491Bacteria
214269Bacteria
4507PlanctomycetesbacteriumADurb.Bin126
17550Gemmatimonadetes
226786candidatedivisionZixibacteriabacterium
28449Desulfomoniletiedjei
706581CandidatusVecturithrixgranuli
307936candidatedivisionKSB3bacterium
91398candidatedivisionKSB3bacterium
47746DesulfobacteralesbacteriumS7086C20
62563Desulfovermiculushalophilus
634068Desulfovermiculushalophilus
342203Desulfobacteraceae
491455Bacteria
164891Bacteria
105532Bacteria
512247Bacteria
314588Bacteria
765412Desulfobacteraceae
170005Bacteria
28505Bacteria

Supplementary Figure 10. Phylogenetic tree based on amino-acid sequences of cold shock protein (*cspA*).

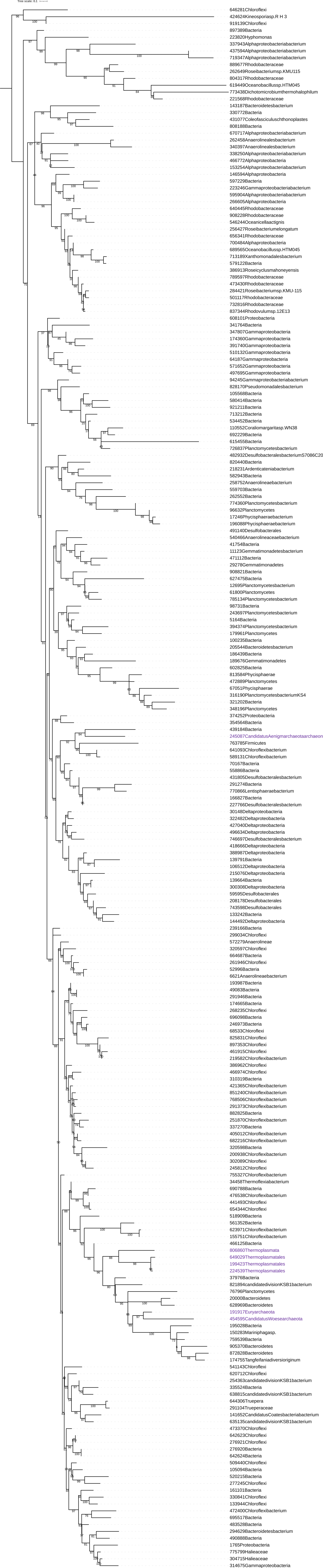
Tree scale: 0.1
646281Chloroflexi
424624Kineosporiasp.R H 3
919139Chloroflexi
897389Bacteria
223820Hyphomonas
337943Alphaproteobacteriabacterium
437594Alphaproteobacteriabacterium
719347Alphaproteobacteriabacterium
889677Rhodobacteraceae
262649Roseibacteriumsp.KMU115
804317Rhodobacteraceae
619449Oceanobacillussp.HTM045
773438Dichotomicrobiumthermohalophilum
221568Rhodobacteraceae
143187Bacteroidetesbacterium
330772Bacteria
431077Coleofasciculuschthonoplastes
808188Bacteria
670717Alphaproteobacteriabacterium
262458Anaerolinealesbacterium
340397Anaerolinealesbacterium
338250Alphaproteobacteriabacterium
466772Alphaproteobacteria
153254Alphaproteobacteriabacterium
146594Alphaproteobacteria
597229Bacteria
223246Gammaproteobacteriabacterium
595904Alphaproteobacteriabacterium
266605Alphaproteobacteria
640445Rhodobacteraceae
908228Rhodobacteraceae
546244Oceanicellaactignis
256427Roseibacteriumelongatum
656341Rhodobacteraceae
700484Alphaproteobacteria
689565Oceanobacillussp.HTM045
713189Xanthomonadalesbacterium
579122Bacteria
386913Roseicyclusmahoneyensis
789597Rhodobacteraceae
473430Rhodobacteraceae
284421Roseibacteriumsp.KMU-115
501117Rhodobacteraceae
732816Rhodobacteraceae
837344Rhodovulumsp.12E13
608101Proteobacteria
341764Bacteria
347807Gammaproteobacteria
174360Gammaproteobacteria
391740Gammaproteobacteria
510132Gammaproteobacteria
64187Gammaproteobacteria
571652Gammaproteobacteria
497695Gammaproteobacteria
94245Gammaproteobacteriabacterium
828170Pseudomonadalesbacterium
105568Bacteria
580414Bacteria
921211Bacteria
713212Bacteria
534452Bacteria
110552Coraliomargaritasp.WN38
692229Bacteria
615455Bacteria
726837Planctomycetesbacterium
482932DesulfobacteralesbacteriumS7086C20
820440Bacteria
218231Ardenticatenabacterium
582943Bacteria
258752Anaerolineaebacterium
559703Bacteria
262552Bacteria
774360Planctomycetesbacterium
96632Planctomycetes
17246Phycisphaerabacterium
196088Phycisphaeraebacterium
491140Desulfobacterales
540466Anaerolineaceaebacterium
41754Bacteria
11123Gemmatimonadetesbacterium
471112Bacteria
29278Gemmatimonadetes
908821Bacteria
627475Bacteria
12695Planctomycetesbacterium
61800Planctomycetes
785134Planctomycetesbacterium
98731Bacteria
243697Planctomycetesbacterium
5164Bacteria
394374Planctomycetesbacterium
179961Planctomycetes
100235Bacteria
205544Bacteroidetesbacterium
186439Bacteria
189676Gemmatimonadetes
602825Bacteria
813584Phycisphaerae
472889Planctomycetes
67051Phycisphaerae
316190PlanctomycetesbacteriumKS4
321202Bacteria
348196Planctomycetes
374252Proteobacteria
354564Bacteria
439184Bacteria
245087CandidatusAenigmarchaeotaarchaeon
763785Firmicutes
641093Chloroflexibacterium
589131Chloroflexibacterium
70167Bacteria
55886Bacteria
431805Desulfobacteralesbacterium
291274Bacteria
770866Lentisphaeraebacterium
166827Bacteria
227766Desulfobacteralesbacterium
30148Deltaproteobacteria
322482Deltaproteobacteria
427040Deltaproteobacteria
496634Deltaproteobacteria
746697Desulfobacteralesbacterium
418666Deltaproteobacteria
388987Deltaproteobacteria
139791Bacteria
106512Deltaproteobacteria
215076Deltaproteobacteria
139664Bacteria
300308Deltaproteobacteria
59595Desulfobacterales
208178Desulfobacterales
743598Desulfobacterales
133242Bacteria
144492Deltaproteobacteria
239166Bacteria
299034Chloroflexi
572279Anaerolineae
320597Chloroflexi
664687Bacteria
261946Chloroflexi
52996Bacteria
6621Anaerolineaebacterium
193987Bacteria
49083Bacteria
291946Bacteria
174665Bacteria
268235Chloroflexi
696098Bacteria
246973Bacteria
68533Chloroflexi
825831Chloroflexi
897353Chloroflexi
461915Chloroflexi
219582Chloroflexibacterium
386962Chloroflexi
466974Chloroflexi
310319Bacteria
421365Chloroflexibacterium
851240Chloroflexibacterium
768506Chloroflexibacterium
291373Chloroflexibacterium
882825Bacteria
251870Chloroflexibacterium
337270Bacteria
405012Chloroflexibacterium
682216Chloroflexibacterium
320598Bacteria
200938Chloroflexibacterium
302089Chloroflexi
245812Chloroflexi
755327Chloroflexibacterium
34458Thermoflexiabacterium
690788Bacteria
476538Chloroflexibacterium
441493Chloroflexi
654344Chloroflexi
518909Bacteria
561352Bacteria
623971Chloroflexibacterium
155751Chloroflexibacterium
466125Bacteria
806860Thermoplasmata
649029Thermoplasmatales
199423Thermoplasmatales
224539Thermoplasmatales
37976Bacteria
821894candidatedivisionKSB1bacterium
76796Planctomycetes
20000Bacteroidetes
628969Bacteroidetes
191917Euryarchaeota
454595CandidatusWoesearchaeota
195028Bacteria
150283Mariniphagasp.
759539Bacteria
905370Bacteroidetes
872828Bacteroidetes
174755Tangfeifaniadiversioriginum
541143Chloroflexi
620712Chloroflexi
254363candidatedivisionKSB1bacterium
335524Bacteria
638815candidatedivisionKSB1bacterium
644306Truepera
291104Trueperaceae
141652CandidatusCoatesbacteriabacterium
635135candidatedivisionKSB1bacterium
473370Chloroflexi
642623Chloroflexi
276921Chloroflexi
276920Bacteria
642624Bacteria
509440Chloroflexi
105094Bacteria
520215Bacteria
277245Chloroflexi
161101Bacteria
330841Chloroflexi
133944Chloroflexi
472400Chloroflexibacterium
695517Bacteria
483528Bacteria
294629Bacteroidetesbacterium
490888Bacteria
1765Proteobacteria
775799Halieaceae
304715Halieaceae
314675Gammaproteobacteria

Supplementary Figure 11. Phylogenetic tree based on amino-acid sequences of phage shock protein C (*pspC*).

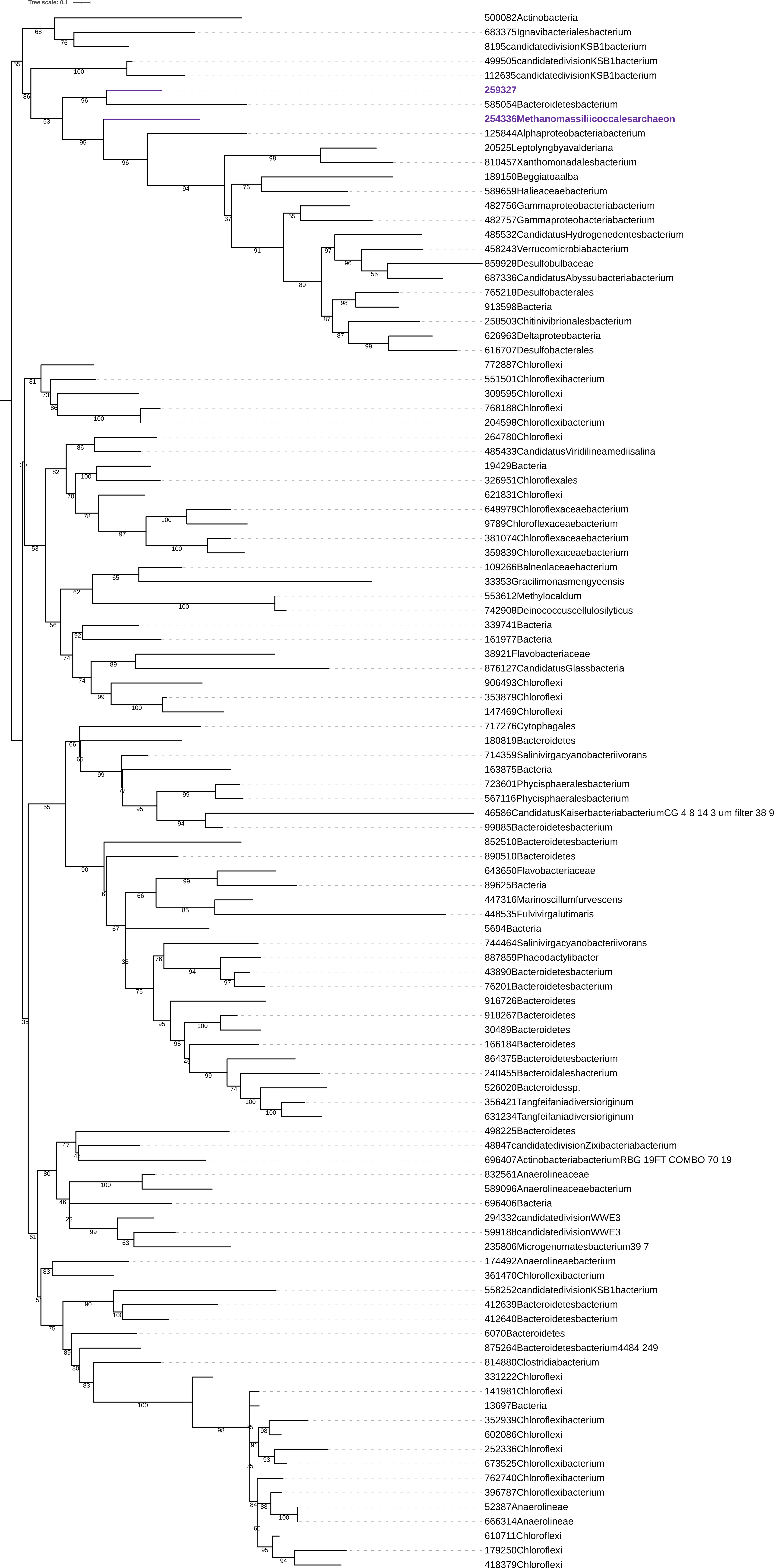

Tree scale: 0.1
500082Actinobacteria
683375Ignavibacterialesbacterium
8195candidatedivisionKSB1bacterium
499505candidatedivisionKSB1bacterium
112635candidatedivisionKSB1bacterium
259327
585054Bacteroidetesbacterium
254336Methanomassiliicoccalesarchaeon
125844Alphaproteobacteriabacterium
20525Leptolyngbyavalderiana
810457Xanthomonadalesbacterium
189150Beggiatoaalba
589659Halieaceaebacterium
482756Gammaproteobacteriabacterium
482757Gammaproteobacteriabacterium
485532CandidatusHydrogenedentesbacterium
458243Verrucomicrobiabacterium
859928Desulfobulbaceae
687336CandidatusAbyssubacteriabacterium
765218Desulfobacterales
913598Bacteria
258503Chitinivibrionalesbacterium
626963Deltaproteobacteria
616707Desulfobacterales
772887Chloroflexi
551501Chloroflexibacterium
309595Chloroflexi
768188Chloroflexi
204598Chloroflexibacterium
264780Chloroflexi
485433CandidatusViridilineamediisalina
19429Bacteria
326951Chloroflexales
621831Chloroflexi
649979Chloroflexaceaebacterium
9789Chloroflexaceaebacterium
381074Chloroflexaceaebacterium
359839Chloroflexaceaebacterium
109266Balneolaceaebacterium
33353Gracilimonasmengyeensis
553612Methylocaldum
742908Deinococcuscellulosilyticus
339741Bacteria
161977Bacteria
38921Flavobacteriaceae
876127CandidatusGlassbacteria
906493Chloroflexi
353879Chloroflexi
147469Chloroflexi
717276Cytophagales
180819Bacteroidetes
714359Salinivirgacyanobacteriivorans
163875Bacteria
723601Phycisphaeralesbacterium
567116Phycisphaeralesbacterium
46586CandidatusKaiserbacteriabacteriumCG 4 8 14 3 um filter 38 9
99885Bacteroidetesbacterium
852510Bacteroidetesbacterium
890510Bacteroidetes
643650Flavobacteriaceae
89625Bacteria
447316Marinoscillumfurvescens
448535Fulvivirgalutimaris
5694Bacteria
744464Salinivirgacyanobacteriivorans
887859Phaeodactylibacter
43890Bacteroidetesbacterium
76201Bacteroidetesbacterium
916726Bacteroidetes
918267Bacteroidetes
30489Bacteroidetes
166184Bacteroidetes
864375Bacteroidetesbacterium
240455Bacteroidalesbacterium
526020Bacteroidessp.
356421Tangfeifaniadiversioriginum
631234Tangfeifaniadiversioriginum
498225Bacteroidetes
48847candidatedivisionZixibacteriabacterium
696407ActinobacteriabacteriumRBG 19FT COMBO 70 19
832561Anaerolineaceae
589096Anaerolineaceaebacterium
696406Bacteria
294332candidatedivisionWWE3
599188candidatedivisionWWE3
235806Microgenomatesbacterium39 7
174492Anaerolineaebacterium
361470Chloroflexibacterium
558252candidatedivisionKSB1bacterium
412639Bacteroidetesbacterium
412640Bacteroidetesbacterium
6070Bacteroidetes
875264Bacteroidetesbacterium4484 249
814880Clostridiabacterium
331222Chloroflexi
141981Chloroflexi
13697Bacteria
352939Chloroflexibacterium
602086Chloroflexi
252336Chloroflexi
673525Chloroflexibacterium
762740Chloroflexibacterium
396787Chloroflexibacterium
52387Anaerolineae
666314Anaerolineae
610711Chloroflexi
179250Chloroflexi
418379Chloroflexi

Supplementary Figure 12. Phylogenetic tree based on amino-acid sequences of excinuclease ABC subunit A (*uvrA*).

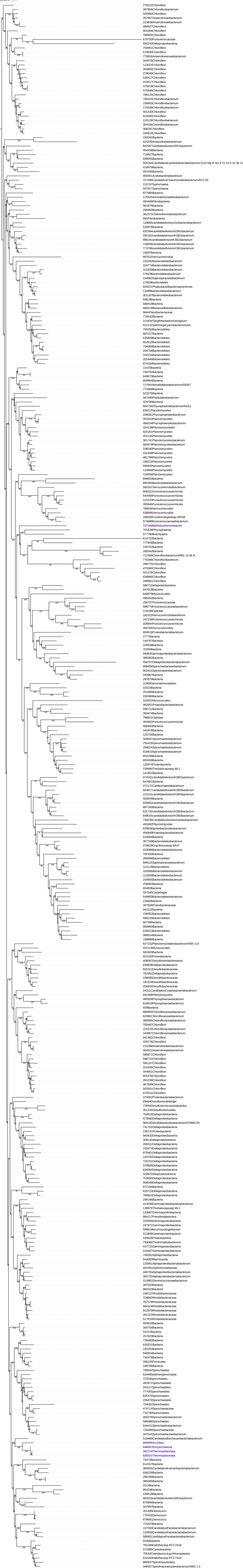

Supplementary Figure 13. Phylogenetic tree based on amino-acid sequences of excinuclease ABC subunit B (*uvrB*).

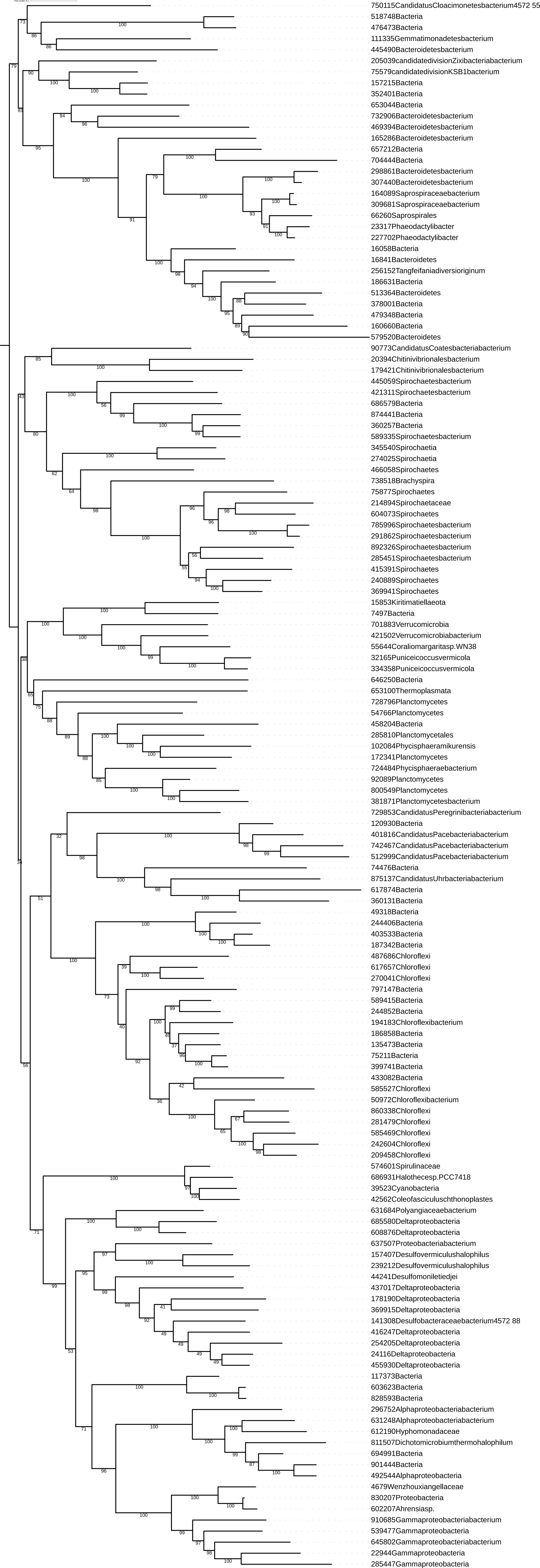
750115CandidatusCloacimonetesbacterium4572 55
518748Bacteria
476473Bacteria
111335Gemmatimonadetesbacterium
445490Bacteroidetesbacterium
205039candidatedivisionZixibacteriabacterium
75579candidatedivisionKSB1bacterium
157215Bacteria
352401Bacteria
653044Bacteria
732906Bacteroidetesbacterium
469394Bacteroidetesbacterium
165286Bacteroidetesbacterium
657212Bacteria
704444Bacteria
298861Bacteroidetesbacterium
307440Bacteroidetesbacterium
164089Saprospiraceaebacterium
309681Saprospiraceaebacterium
66260Saprospirales
23317Phaeodactylibacter
227702Phaeodactylibacter
16058Bacteria
16841Bacteroidetes
256152Tangfeifaniadiversioriginum
186631Bacteria
513364Bacteroidetes
378001Bacteria
479348Bacteria
160660Bacteria
579520Bacteroidetes
90773CandidatusCoatesbacteriabacterium
20394Chitinivibrionalesbacterium
179421Chitinivibrionalesbacterium
445059Spirochaetesbacterium
421311Spirochaetesbacterium
686579Bacteria
874441Bacteria
360257Bacteria
589335Spirochaetesbacterium
345540Spirochaetia
274025Spirochaetia
466058Spirochaetes
738518Brachyspira
75877Spirochaetes
214894Spirochaetaceae
604073Spirochaetes
785996Spirochaetesbacterium
291862Spirochaetesbacterium
892326Spirochaetesbacterium
285451Spirochaetesbacterium
415391Spirochaetes
240889Spirochaetes
369941Spirochaetes
15853Kiritimatiellaeota
7497Bacteria
701883Verrucomicrobia
421502Verrucomicrobiabacterium
55644Coraliomargaritasp.WN38
32165Puniceicoccusvermicola
334358Puniceicoccusvermicola
646250Bacteria
653100Thermoplasmata
728796Planctomycetes
54766Planctomycetes
458204Bacteria
285810Planctomycetales
102084Phycisphaeramikurensis
172341Planctomycetes
724484Phycisphaeraebacterium
92089Planctomycetes
800549Planctomycetes
381871Planctomycetesbacterium
729853CandidatusPeregrinibacteriabacterium
120930Bacteria
401816CandidatusPacebacteriabacterium
742467CandidatusPacebacteriabacterium
512999CandidatusPacebacteriabacterium
74476Bacteria
875137CandidatusUhrbacteriabacterium
617874Bacteria
360131Bacteria
49318Bacteria
244406Bacteria
403533Bacteria
187342Bacteria
487686Chloroflexi
617657Chloroflexi
270041Chloroflexi
797147Bacteria
589415Bacteria
244852Bacteria
194183Chloroflexibacterium
186858Bacteria
135473Bacteria
75211Bacteria
399741Bacteria
433082Bacteria
585527Chloroflexi
50972Chloroflexibacterium
860338Chloroflexi
281479Chloroflexi
585469Chloroflexi
242604Chloroflexi
209458Chloroflexi
574601Spirulinaceae
686931Halothecesp.PCC7418
39523Cyanobacteria
42562Coleofasciculuschthonoplastes
631684Polyangiaceaebacterium
685580Deltaproteobacteria
608876Deltaproteobacteria
637507Proteobacteriabacterium
157407Desulfovermiculushalophilus
239212Desulfovermiculushalophilus
44241Desulfomoniletiedjei
437017Deltaproteobacteria
178190Deltaproteobacteria
369915Deltaproteobacteria
141308Desulfobacteraceaebacterium4572 88
416247Deltaproteobacteria
254205Deltaproteobacteria
24116Deltaproteobacteria
455930Deltaproteobacteria
117373Bacteria
603623Bacteria
828593Bacteria
296752Alphaproteobacteriabacterium
631248Alphaproteobacteriabacterium
612190Hyphomonadaceae
811507Dichotomicrobiumthermohalophilum
694991Bacteria
901444Bacteria
492544Alphaproteobacteria
4679Wenzhouxiangellaceae
830207Proteobacteria
602207Ahrensiasp.
910685Gammaproteobacteriabacterium
539477Gammaproteobacteria
645802Gammaproteobacteriabacterium
22944Gammaproteobacteria
285447Gammaproteobacteria

Supplementary Figure 14. Phylogenetic tree based on amino-acid sequences of excinuclease ABC subunit C (*uvrC*).

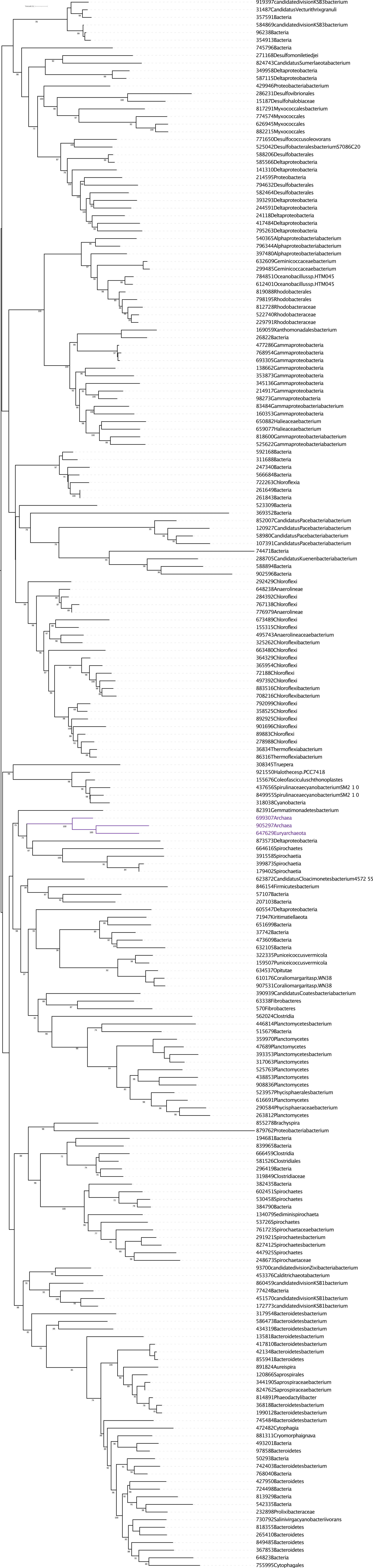
919397candidatedivisionKSB3bacterium
31487CandidatusVecturithrixgranuli
357591Bacteria
584869candidatedivisionKSB3bacterium
96238Bacteria
354913Bacteria
745796Bacteria
271168Desulfomoniletiedjei
824743CandidatusSumerlaeotabacterium
349958Deltaproteobacteria
587115Deltaproteobacteria
429946Proteobacteriabacterium
286231Desulfovibrionales
15187Desulfohalobiaceae
817291Myxococcalesbacterium
774574Myxococcales
626945Myxococcales
882215Myxococcales
771650Desulfococcusoleovorans
525042DesulfobacteralesbacteriumS7086C20
588206Desulfobacterales
585566Deltaproteobacteria
141310Deltaproteobacteria
214595Proteobacteria
794632Desulfobacterales
582464Desulfobacterales
393293Deltaproteobacteria
244591Deltaproteobacteria
24118Deltaproteobacteria
417484Deltaproteobacteria
795263Deltaproteobacteria
540365Alphaproteobacteriabacterium
796344Alphaproteobacteriabacterium
397480Alphaproteobacteriabacterium
632609Geminicoccaceaebacterium
299485Geminicoccaceaebacterium
784851Oceanobacillussp.HTM045
612401Oceanobacillussp.HTM045
819088Rhodobacterales
798195Rhodobacterales
812728Rhodobacteraceae
522740Rhodobacteraceae
229791Rhodobacteraceae
169059Xanthomonadalesbacterium
26822Bacteria
477286Gammaproteobacteria
768954Gammaproteobacteria
693305Gammaproteobacteria
138662Gammaproteobacteria
353873Gammaproteobacteria
345136Gammaproteobacteria
214917Gammaproteobacteria
98273Gammaproteobacteria
83484Gammaproteobacteriabacterium
160353Gammaproteobacteria
650882Halieaceaebacterium
659077Halieaceaebacterium
818600Gammaproteobacteriabacterium
525622Gammaproteobacteriabacterium
592168Bacteria
311688Bacteria
247340Bacteria
566684Bacteria
722263Chloroflexia
261649Bacteria
261843Bacteria
523309Bacteria
369352Bacteria
852007CandidatusPacebacteriabacterium
120927CandidatusPacebacteriabacterium
58980CandidatusPacebacteriabacterium
107391CandidatusPacebacteriabacterium
74471Bacteria
288705CandidatusKuenenbacteriabacterium
588894Bacteria
902596Bacteria
292429Chloroflexi
648238Anaerolineae
284392Chloroflexi
767138Chloroflexi
776979Anaerolineae
673489Chloroflexi
155315Chloroflexi
495743Anaerolineaceaebacterium
325262Chloroflexibacterium
663480Chloroflexi
364329Chloroflexi
365954Chloroflexi
72188Chloroflexi
497392Chloroflexi
883516Chloroflexibacterium
708216Chloroflexibacterium
792099Chloroflexi
358525Chloroflexi
892925Chloroflexi
901696Chloroflexi
89883Chloroflexi
278988Chloroflexi
36834Thermoflexiabacterium
86316Thermoflexiabacterium
308345Truepera
921550Halothecesp.PCC7418
155676Coleofasciculuschthonoplastes
437656SpirulinaceaecyanobacteriumSM2 1 0
849955SpirulinaceaecyanobacteriumSM2 1 0
318038Cyanobacteria
82391Gemmatimonadetesbacterium
699307Archaea
905297Archaea
647629Euryarchaeota
873573Deltaproteobacteria
664616Spirochaetes
391558Spirochaetia
399873Spirochaetia
179402Spirochaetia
623872CandidatusCloacimonetesbacterium4572 55
846154Firmicutesbacterium
57107Bacteria
207103Bacteria
605547Deltaproteobacteria
71947Kiritimatiellaeota
651699Bacteria
37742Bacteria
473609Bacteria
632105Bacteria
322335Puniceicoccusvermicola
159507Puniceicoccusvermicola
634537Opitutae
610176Coraliomargaritasp.WN38
907531Coraliomargaritasp.WN38
390939CandidatusCoatesbacteriabacterium
63338Fibrobacteres
570Fibrobacteres
562024Clostridia
446814Planctomycetesbacterium
515679Bacteria
359970Planctomycetes
47689Planctomycetes
393353Planctomycetesbacterium
317063Planctomycetes
525763Planctomycetes
438853Planctomycetes
908836Planctomycetes
523957Phycisphaeralesbacterium
616691Planctomycetes
290584Phycisphaeraceaebacterium
263812Planctomycetes
855278Brachyspira
879762Proteobacteriabacterium
194681Bacteria
839965Bacteria
666459Clostridia
581526Clostridiales
296419Bacteria
319849Clostridiaceae
382435Bacteria
602451Spirochaetes
530458Spirochaetes
384790Bacteria
134079Sediminispirochaeta
53726Spirochaetes
761723Spirochaetaceaebacterium
291921Spirochaetesbacterium
827412Spirochaetesbacterium
447925Spirochaetes
248673Spirochaetaceae
93700candidatedivisionZixibacteriabacterium
453376Calditrichaeotabacterium
860459candidatedivisionKSB1bacterium
77424Bacteria
451570candidatedivisionKSB1bacterium
172773candidatedivisionKSB1bacterium
317954Bacteroidetesbacterium
586473Bacteroidetesbacterium
434319Bacteroidetesbacterium
13581Bacteroidetesbacterium
417810Bacteroidetesbacterium
42134Bacteroidetesbacterium
855941Bacteroidetes
891824Aureispira
120866Saprospirales
344190Saprospiraceaebacterium
824762Saprospiraceaebacterium
814891Phaeodactylibacter
36818Bacteroidetesbacterium
199012Bacteroidetesbacterium
745484Bacteroidetesbacterium
472482Cytophagia
881311Cryomorphaignava
493201Bacteria
97858Bacteroidetes
50293Bacteria
742403Bacteroidetesbacterium
768040Bacteria
427950Bacteroidetes
724498Bacteroidetes
813929Bacteria
542335Bacteria
232898Prolixibacteraceae
730792Salinivirgacyanobacteriivorans
818355Bacteroidetes
265410Bacteroidetes
849485Bacteroidetes
367853Bacteroidetes
64823Bacteria
755995Cytophagales

Supplementary Figure 15. Phylogenetic tree based on amino-acid sequences of DNA helicase II ATP dependent DNA helicase (*uvrD, pcrA*).

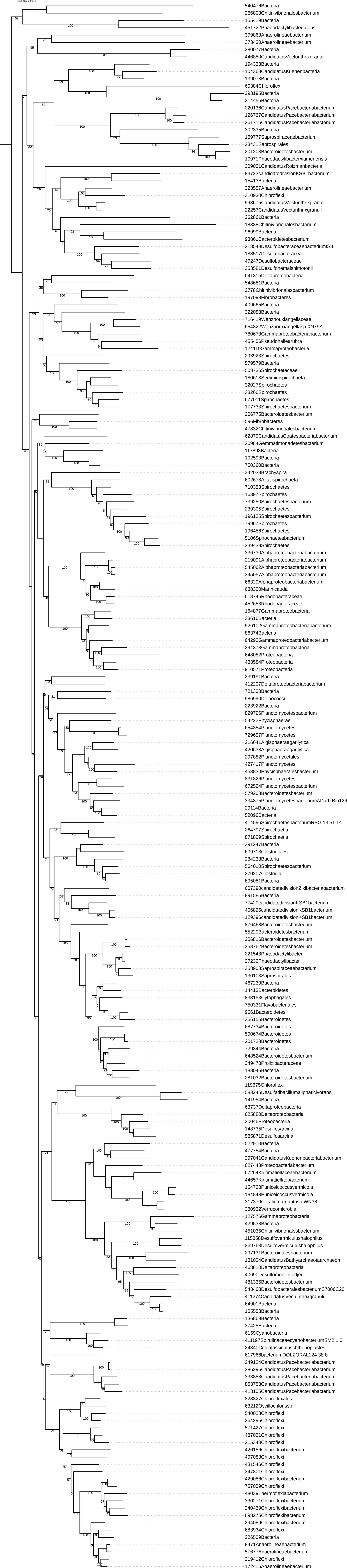
Tree scale: 0.1
540476Bacteria
266808Chitinivibrionalesbacterium
155419Bacteria
451722Phaeodactylibacterluteus
379868Anaerolineaebacterium
373430Anaerolineaebacterium
280077Bacteria
446850CandidatusVecturithrixgranuli
194333Bacteria
104363CandidatusKuenenbacteria
139078Bacteria
60384Chloroflexi
293195Bacteria
214455Bacteria
220136CandidatusPacebacteriabacterium
126767CandidatusPacebacteriabacterium
261716CandidatusPacebacteriabacterium
302335Bacteria
169777Saprospiraceaebacterium
23431Saprospirales
201203Bacteroidetesbacterium
10971Phaeodactylibacterxiamenensis
309031CandidatusRoizmanbacteria
83723candidatedivisionKSB1bacterium
15413Bacteria
323557Anaerolineaebacterium
310930Chloroflexi
593675CandidatusVecturithrixgranuli
22257CandidatusVecturithrixgranuli
262861Bacteria
18338Chitinivibrionalesbacterium
96999Bacteria
93861Bacteroidetesbacterium
218548DesulfobacteraceaebacteriumIS3
188517Desulfobacteraceae
47247Desulfobacteraceae
353581Desulfonemaishimotonii
641315Deltaproteobacteria
548681Bacteria
2778Chitinivibrionalesbacterium
197093Fibrobacteres
409665Bacteria
322088Bacteria
716419Wenzhouxiangellaceae
654822Wenzhouxiangellasp.XN79A
780678Gammaproteobacteriabacterium
455456Pseudohaliea rubra
124119Gammaproteobacteria
293923Spirochaetes
579579Bacteria
508736Spirochaetaceae
180618Sediminispirochaeta
32027Spirochaetes
33266Spirochaetes
677011Spirochaetes
177733Spirochaetesbacterium
206775Bacteroidetesbacterium
596Fibrobacteres
47832Chitinivibrionalesbacterium
62879CandidatusCoatesbacteriabacterium
20984Gemmatimonadetesbacterium
117893Bacteria
102593Bacteria
750360Bacteria
342038Brachyspira
602678Alkalispirochaeta
710358Spirochaetes
16397Spirochaetes
739280Spirochaetesbacterium
239395Spirochaetes
196125Spirochaetesbacterium
79967Spirochaetes
196456Spirochaetes
5106Spirochaetesbacterium
339439Spirochaetes
336730Alphaproteobacteriabacterium
219091Alphaproteobacteriabacterium
545062Alphaproteobacteriabacterium
345057Alphaproteobacteriabacterium
66329Alphaproteobacteriabacterium
638320Marinicauda
618746Rhodobacteraceae
452653Rhodobacteraceae
164877Gammaproteobacteria
33616Bacteria
526102Gammaproteobacteriabacterium
86374Bacteria
64292Gammaproteobacteriabacterium
294373Gammaproteobacteria
648082Proteobacteria
433584Proteobacteria
910571Proteobacteria
239191Bacteria
412207Deltaproteobacteriabacterium
721308Bacteria
586990Deinococci
223922Bacteria
829796Planctomycetesbacterium
54222Phycisphaerae
654354Planctomycetes
729657Planctomycetes
216641Algisphaeraagarilytica
420638Algisphaeraagarilytica
297882Planctomycetales
427417Planctomycetes
453830Phycisphaeralesbacterium
831826Planctomycetes
672524Planctomycetesbacterium
579203Bacteroidetesbacterium
334875PlanctomycetesbacteriumADurb.Bin126
29114Bacteria
52096Bacteria
414596SpirochaetesbacteriumRBG 13 51 14
264797Spirochaetia
871809Spirochaetia
391247Bacteria
609713Clostridiales
284238Bacteria
564010Spirochaetesbacterium
270207Clostridia
695081Bacteria
607390candidatedivisionZixibacteriabacterium
891585Bacteria
77420candidatedivisionKSB1bacterium
406825candidatedivisionKSB1bacterium
129396candidatedivisionKSB1bacterium
876468Bacteroidetesbacterium
55220Bacteroidetesbacterium
256816Bacteroidetesbacterium
358762Bacteroidetesbacterium
221548Phaeodactylibacter
27230Phaeodactylibacter
358903Saprospiraceaebacterium
130103Saprospirales
467239Bacteria
14413Bacteroidetes
833153Cytophagales
750331Flavobacteriales
9661Bacteroidetes
356156Bacteroidetes
687734Bacteroidetes
590674Bacteroidetes
201728Bacteroidetes
729344Bacteria
648524Bacteroidetesbacterium
349478Prolixibacteraceae
188046Bacteria
281032Bacteroidetesbacterium
119675Chloroflexi
583245Desulfatibacillumaliphaticivorans
141954Bacteria
63737Deltaproteobacteria
625880Deltaproteobacteria
30046Proteobacteria
148735Desulfosarcina
585871Desulfosarcina
522910Bacteria
477754Bacteria
297041CandidatusKuenenbacteriabacterium
627448Proteobacteriabacterium
67264Kiritimatiellaeaebacterium
44657Kiritimatiellaebacterium
154728Puniceicoccusvermicola
184843Puniceicoccusvermicola
317370Coraliomargaritasp.WN38
380932Verrucomicrobia
127576Gammaproteobacteria
429538Bacteria
451035Chitinivibrionalesbacterium
115358Desulfovermiculushalophilus
269763Desulfovermiculushalophilus
297131Bacteroidalesbacterium
181004CandidatusBathyarchaeotaarchaeon
468810Deltaproteobacteria
40690Desulfomonilétiedjei
481335Bacteroidetesbacterium
543468DesulfobacteralesbacteriumS7086C20
411274CandidatusVecturithrixgranuli
64901Bacteria
155553Bacteria
136869Bacteria
37425Bacteria
6159Cyanobacteria
411197SpirulinaceaecyanobacteriumSM2 1 0
24340Coleofasciculuschthonoplastes
617966bacteriumDOLZORAL124 38 8
249124CandidatusPacebacteriabacterium
286295CandidatusPacebacteriabacterium
333888CandidatusPacebacteriabacterium
863753CandidatusPacebacteriabacterium
413105CandidatusPacebacteriabacterium
828327Chloroflexales
63212Oscillochlorissp.
540028Chloroflexi
264296Chloroflexi
571427Chloroflexi
487031Chloroflexi
215340Chloroflexi
426156Chloroflexibacterium
497083Chloroflexi
431546Chloroflexi
347801Chloroflexi
429086Chloroflexibacterium
757059Chloroflexi
48039Thermoflexibacterium
330271Chloroflexibacterium
240439Chloroflexibacterium
698275Chloroflexibacterium
294089Chloroflexibacterium
683934Chloroflexi
226509Bacteria
8471Anaerolineaebacterium
57677Anaerolineaebacterium
219412Chloroflexi
172415Anaerolineaebacterium
